# Solvent Impact on the Properties of Benchmark Metal–Organic Frameworks: Acetonitrile‐Based Synthesis of CAU‐10, Ce‐UiO‐66, and Al‐MIL‐53

**DOI:** 10.1002/chem.201905376

**Published:** 2020-03-09

**Authors:** Sebastian Leubner, Robert Stäglich, Julia Franke, Jannick Jacobsen, Jonas Gosch, Renée Siegel, Helge Reinsch, Guillaume Maurin, Jürgen Senker, Pascal G. Yot, Norbert Stock

**Affiliations:** ^1^ Department for Inorganic Chemistry University of Kiel Max-Eyth Strasse 2 24118 Kiel Germany; ^2^ Inorganic Chemistry III University of Bayreuth Universitätsstrasse 30 95447 Bayreuth Germany; ^3^ Institut Charles Gerhard Montpellier (ICGM) UMR 5253 Université de Montpellier, CNRS ENSCM, CC 1505 Place Eugène Bataillon 43095 Montpellier cedex 05 France

**Keywords:** adsorption, metal–organic frameworks, microporous materials, NMR spectroscopy, solvent effects

## Abstract

Herein is reported the utilization of acetonitrile as a new solvent for the synthesis of the three significantly different benchmark metal–organic frameworks (MOFs) CAU‐10, Ce‐UiO‐66, and Al‐MIL‐53 of idealized composition [Al(OH)(ISO)], [Ce_6_O_4_(OH)_4_(BDC)_6_], and [Al(OH)(BDC)], respectively (ISO^2−^: isophthalate, BDC^2−^: terephthalate). Its use allowed the synthesis of Ce‐UiO‐66 on a gram scale. While CAU‐10 and Ce‐UiO‐66 exhibit properties similar to those reported elsewhere for these two materials, the obtained Al‐MIL‐53 shows no structural flexibility upon adsorption of hydrophilic or hydrophobic guest molecules such as water and xenon and is stabilized in its large‐pore form over a broad temperature range (130–450 K). The stabilization of the large‐pore form of Al‐MIL‐53 was attributed to a high percentage of noncoordinating −COOH groups as determined by solid‐state NMR spectroscopy. The defective material shows an unusually high water uptake of 310 mg g^−1^ within the range of 0.45 to 0.65 p/p°. In spite of showing no breathing effect upon water adsorption it exhibits distinct mechanical properties. Thus, mercury intrusion porosimetry studies revealed that the solid can be reversibly forced to breathe by applying moderate pressures (≈60 MPa).

## Graphical abstract

**Metal–organic frameworks**: CAU‐10, Ce‐UiO‐66, and Al‐MIL‐53 were synthesized by utilizing acetonitrile as the solvent. The typical breathing behavior of Al‐MIL‐53 upon guest molecule adsorption was inhibited by a high concentration of defects. As shown by NMR spectroscopy these consist of noncoordinated −COOH groups. Breathing could be enforced by applying a pressure of about 60 MPa during mercury intrusion.

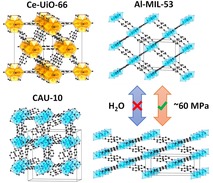

## Introduction

Over the past years metal–organic frameworks (MOFs) have gained much attention because of their potential applications in, for example, gas storage^1^ and separation,^2^ sensing,^3^ catalysis,^4^ heat transformation,^5^ and the medical sector.^6^ This led to intense research activities on the development of new and synthetically challenging MOFs with in some cases highly complex structures.^7^ Nowadays, applicability, sustainability, and simplicity of the syntheses are coming more and more into the focus of interest, because the transition between the exploration of new MOFs and application‐oriented research needs to be realized.

One approach to create more sustainable synthesis routes is to replace hazardous and environmentally unfriendly solvents by less harmful ones, basically following the twelve principles of green chemistry.^8^ In common MOF syntheses often aprotic and highly polar solvents are required to dissolve organic molecules (linkers) in sufficient amounts. Unfortunately, these solvents (a prominent example is *N*,*N*‐dimethylformamide (DMF)) pose many risks regarding safety, occupational health, and environmental impact.^9^

In some selected cases, water has already been used as an alternative solvent, considering for example the synthesis of −COOH‐functionalized Zr‐UiO‐66,^10^ Al‐MIL‐53,^11^ or CAU‐10.^12^ Nonetheless, water‐based routes are often either limited to very polar organic linker molecules or require high temperatures to realize sufficient dissolution of less polar reactants. For solvents in general, harmfulness is often linked to their beneficial properties.9a The use of acetonitrile has been only very little explored in solvothermal MOF syntheses.^13^ Acetonitrile is commercially mainly produced in the Sohio process through catalytic ammoxidation of propene.^14^ Hydrogen cyanide and acetonitrile occur as byproducts. Acetonitrile has similar solvent properties as those of DMF, which are desired in MOF syntheses, while being considered as less hazardous. A major concern regarding DMF is its reproductive toxicity. It is common for amides and not observed for acetonitrile.9a Both solvents are covered and rated by multiple solvent selection guidelines comprising a large number of different criteria. In 2014, Prat et al. published a survey of solvent selection guides, which combined all previous guides in one comprehensive guide with improved consensus.9b In Table 5 of reference 9b, acetonitrile is still listed as a problematic solvent and cannot be considered as fully green, but it is one of the least hazardous options for aprotic polar solvents to date and capable of dissolving smaller organic molecules with low polarity in sufficient amounts.

**Figure 1:**
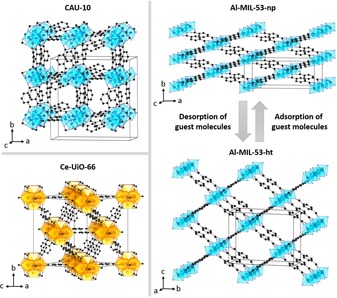
Structures of CAU‐10^18^ (top left), Ce‐UiO‐6616a (bottom left), as well as narrow‐ (top right) and open‐pore form (bottom right) of standard Al‐MIL‐53^11^ with unit cell edges. AlO_6_ octahedra are shown in light blue and Ce_6_O_32_ polyhedra in light orange. Hydrogen atoms are not displayed.

As a typical illustration, an acetonitrile‐based synthetic strategy was applied by using different metal ions and linker molecules (Table S2). On the basis of our previous work on Al‐ and Ce‐MOFs, the following three compounds were chosen for a more detailed study: 1) CAU‐10 [Al(OH)(ISO)] is an aluminum MOF, well‐known for its applicability in adsorption‐driven chilling (ISO^2−^: isophthalate).^12^ It exhibits infinite helical chains of *cis* corner‐sharing AlO_6_ polyhedra, which are interconnected by V‐shaped isophthalate ions to form a three‐dimensional network with square‐shaped, sinusoidal pore channels (Figure 1, top left).^15^ 2) Ce‐UiO‐66 [Ce_6_O_4_(OH)_4_(BDC)_6_] represents the redox‐active^16^ cerium‐based analogue of Zr‐UiO‐66^17^ and contains hexanuclear cerium‐oxygen clusters ([Ce_6_O_4_(OH)_4_]^12+^) (BDC^2−^: terephthalate). Each cluster is coordinated to 12 other clusters via terephthalate ions, which leads to a fcu topology (Figure 1, bottom left).16a 3) Al‐MIL‐53 [Al(OH)(BDC)], a MOF composed of infinite chains of *trans* corner‐sharing AlO_6_ octahedra that are interconnected by terephthalate ions, represents a special type of framework. Its wine‐rack structure has the ability to undergo reversible phase transitions upon adsorption of guest molecules or temperature change (Figure 1, right).^11^, ^19^ This behavior is called “breathing effect”. When, for example, water molecules are adsorbed onto the 1D pore channels of the activated/empty structure (denoted as ht form, ht: high temperature; also known as open or large‐pore form), they create strong hydrogen bonds with bridging OH groups of the inorganic building unit (IBU). These interactions force the framework to contract into its narrow‐pore (np)/low‐temperature (lt) form (also known as closed‐pore (cp) form if no guest molecules are present). The process is reversible upon water desorption.^11^ This breathing behavior has also been observed under mechanical pressure, which makes this material and its isoreticular analogues promising shock absorbers.^20^

Here, we present the syntheses of CAU‐10,^12^, ^15^ Ce‐UiO‐66,16a and Al‐MIL‐53^11^ from acetonitrile as well as the characterization of the products with a special focus on changes of the framework flexibility of Al‐MIL‐53.

## Results and Discussion

The screening of different metal ions and linker molecules using acetonitrile as the solvent resulted in the formation of well‐known MOFs (Table S2, Figures S7 and S8). For a detailed study the compounds CAU‐10, Ce‐UiO‐66, and Al‐MIL‐53 were selected. The solvothermal reaction of aluminum nitrate nonahydrate, Al(NO_3_)_3_ **⋅**9 H_2_O, with nearly insoluble isophthalic or terephthalic acid in acetonitrile at 130 °C for 23 h yielded CAU‐10 or Al‐MIL‐53, respectively. For Ce‐UiO‐66 a milder synthesis route is feasible. Thus, terephthalic acid was reacted with aqueous cerium ammonium nitrate, (NH_4_)_2_[Ce(NO_3_)_6_], solution in acetonitrile at 100 °C for 2 h under reflux. It is remarkable that the reaction time for Ce‐UiO‐66 could be increased to 2 h, because DMF‐based syntheses for this material are commonly limited to very short reaction times of about 15 to 30 min. At longer reaction times, DMF decomposition to dimethylamine and formic acid becomes dominant and the thermodynamically favored product, cerium(III) formate, Ce(O_2_CH)_3_, is formed.16a, 21 This also limits the scalability of the synthesis, and usually only small quantities of Ce‐MOFs have been obtained so far. Thus, by utilizing acetonitrile as the solvent the reaction could be carried out at the 240 mL scale, which resulted in a yield of more than 7 g of this compound.

All as‐synthesized (as) compounds are not yet pure, which is due to only partially reacted metal species or small amounts of linker residues. Additionally, acetamide occurs as a minor impurity. It is the intermediate of the acetonitrile hydrolysis, which ultimately leads to the formation of acetic acid and ammonia. The latter manifests itself through pressure build‐up in the reaction vessel. The hydrolysis is enabled by water, which originates from hydration water or the solvent, as well as catalytically active Lewis acidic metal ions, Al^3+^ or Ce^4+^.^22^ One should keep in mind that the final hydrolysis product, acetic acid, possibly acts as a modulator during nucleation and crystal growth of the title compounds.^23^ However, soluble metal species represent the largest part of impurities and can therefore be easily removed through solvent treatment by using DMF, acetone, ethanol, or water.

Ce‐UiO‐66 (as) was treated with DMF in order to remove unreacted terephthalic acid. Subsequent solvent exchange with acetone finally yields a slightly defective product (section Thermogravimetric and Elemental Analysis in the Supporting Information). Because of smaller amounts of impurities, water treatment is sufficient for CAU‐10 (as), whereas Al‐MIL‐53 (as) requires preliminary washing with ethanol. Otherwise X‐ray amorphous aluminum hydroxide is formed, which complicates the purification process significantly. Detailed descriptions of the synthesis and purification procedures of the three title compounds are given in the Supporting Information (section Synthetic Procedures).

**Figure 2:**
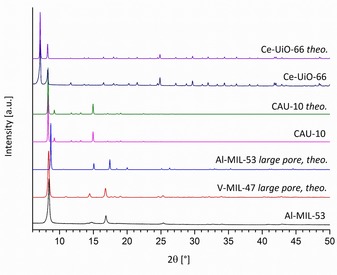
PXRD patterns of Al‐MIL‐53, CAU‐10, and Ce‐UiO‐66 in comparison with their theoretical patterns.^11^, 16a, 18 Al‐MIL‐53 is further compared to the theoretical pattern of large‐pore V‐MIL‐47^24^ in order to emphasize their similarity.

After purification, all products were characterized by powder X‐ray diffraction (PXRD) (Figure 2) and their cell parameters were determined by LeBail fits (Table S3).

The PXRD patterns of CAU‐10 and Ce‐UiO‐66 are well in line with their theoretical patterns. The comparison of the PXRD pattern of Al‐MIL‐53 with the theoretical patterns of standard Al‐MIL‐53 and V‐MIL‐47 [VO(BDC)] in their large‐pore forms reveals that the synthesized product exhibits a structure that is rather reminiscent of the latter. The structural similarity with V‐MIL‐47 is underlined by the corresponding LeBail fit, carried out starting from the cell parameters of V‐MIL‐47 (Figure S4), which shows a good match of the calculated and reported cell parameters (Table S3).

Compositional analysis by thermogravimetric and elemental analyses as well as infrared (IR) spectroscopy and nitrogen adsorption experiments confirm the successful synthesis of CAU‐10, Ce‐UiO‐66, and Al‐MIL‐53. Details are given in the Supporting Information. The micropore volumes and specific surface areas as determined by the Brunauer–Emmett–Teller (BET) method from nitrogen adsorption isotherms by utilizing the approach reported by Rouquerol et al.^25^ are summarized in Table 1 and compared to values previously reported in the literature.

**Table 1:** Results of the N_2_ sorption measurements comparing micropore volumes and BET areas of the title compounds with previously reported values. All micropore volumes were determined at p/p°=0.5 except for those of Al‐MIL‐53‐ht and MIL‐53‐is that were determined at p/p°=0.6.

Material	*a*_S,BET_ (exp.) [m^2^ g^−1^]	*V*_mic_ (exp.) [cm^3^ g^−1^]	*a*_S,BET_ (lit.) [m^2^ g^−1^]	*V*_mic_ (lit.) [cm^3^ g^−1^]
CAU‐10	644	0.27	635^15^	0.25^15^
Ce‐UiO‐66	1207	0.49	128216a	0.5016a
Al‐MIL‐53	1183	0.46	–	–
Al‐MIL‐53‐ht	–	–	1510^26^	0.57^26^
MIL‐53‐is	–	–	1228^26^	0.56^26^

The Al‐MIL‐53 of this work is compared to standard Al‐MIL‐53‐ht and to a nonbreathing MIL‐53 analogue (MIL‐53‐is, is: imidazolium salt).^26^ The BET areas of Al‐MIL‐53 and MIL‐53‐is are similar and significantly lower than those reported for conventional Al‐MIL‐53‐ht. The lower BET area for MIL‐53‐is was attributed to the presence of γ‐AlO(OH), which also stabilizes its large‐pore form and thus inhibits its breathing.^26^ For the present Al‐MIL‐53, the PXRD and spectroscopic analysis did not yield experimental evidence for such an impurity.

**Figure 3:**
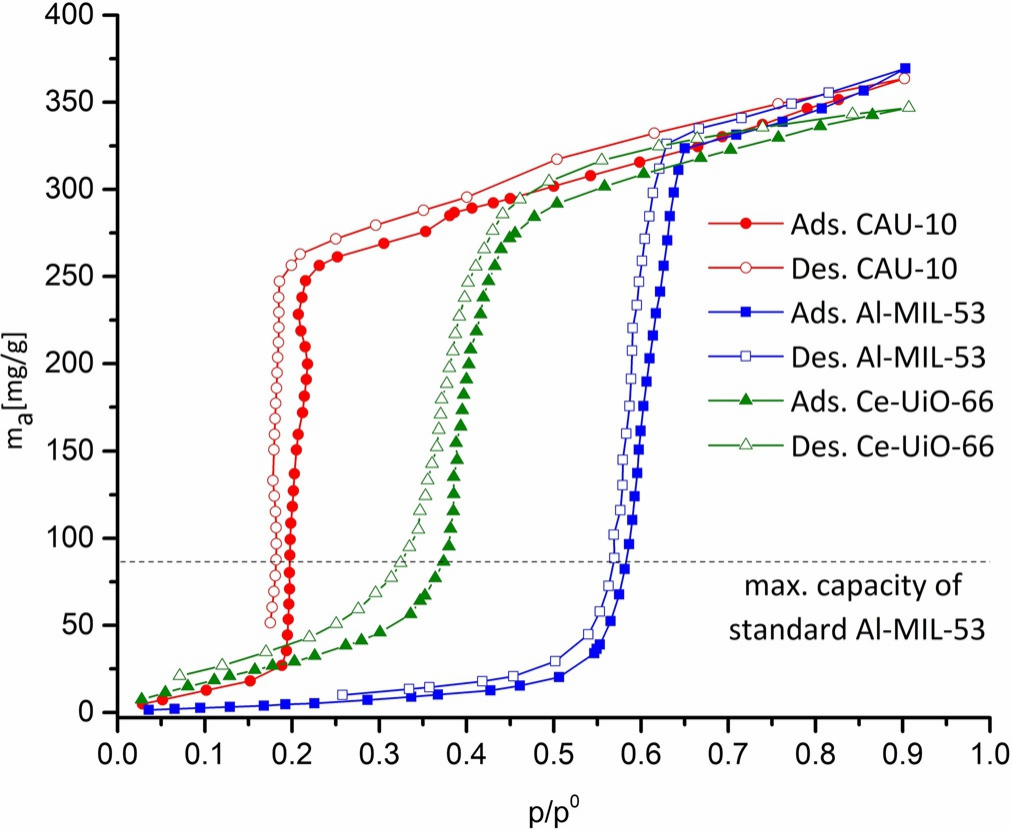
Water adsorption isotherms of CAU‐10, Al‐MIL‐53, and Ce‐UiO‐66. The horizontal black dashed line represents the maximum water capacity of standard Al‐MIL‐53 (90 mg g^−1^) as reported by the Kitagawa group.^27^ The measured maximum capacities for the title compounds are 360 mg g^−1^ (CAU‐10), 360 mg g^−1^ (Al‐MIL‐53), and 340 mg g^−1^ (Ce‐UiO‐66).

Water adsorption experiments (Figure 3) show that the synthesized CAU‐10 exhibits properties similar to those previously reported for this material with a steep increase at p/p°=0.2.^12^ For Ce‐UiO‐66 a one‐step water adsorption (250 mg g^−1^) between p/p° values of 0.25 and 0.5 is observed, which matches data from conventionally synthesized Ce‐UiO‐6616a (Figure S21). In contrast, the Al‐MIL‐53 of this work is more hydrophobic than standard Al‐MIL‐53^27^ and shows a significantly increased one‐step water uptake of 310 mg g^−1^ in the range p/p°=0.45–0.65. Standard Al‐MIL‐53 is limited to a maximum water adsorption capacity of 90 mg g^−1^ because of its pore contraction (breathing) upon water adsorption, and its major uptake lies within the range p/p°=0.2–0.4.^27^ Therefore, the unexpected behavior of the synthesized Al‐MIL‐53 can be related to the absence of such a breathing effect. This is also in line with the hydrophobic character of the material, because the hydrophobic phenyl rings of the linkers are much better accessible for guest molecules when the large‐pore form is retained.^11^, ^28^ It is noteworthy that the adsorption and desorption branch lie within the range (45 to 65 %) recommended by ASHRAE (American Society of Heating, Refrigerating, and Air Conditioning Engineers) for relative indoor humidity of occupied spaces.^29^ Together with the materials’ comparably high capacity in the given range it makes it a potential candidate for indoor moisture control applications.^29^ It could be most preferably used to effectively stabilize moisture levels between 55 and 65 % relative humidity. To further understand and evaluate the material properties, detailed solid‐state NMR spectroscopic measurements and Hg intrusion porosimetry studies were carried out.

^1^H, ^13^C, ^27^Al high‐resolution solid‐state NMR spectroscopic experiments suggest an unusually high defect concentration within the framework, which most probably inhibits the breathing behavior in the present case.

**Figure 4:**
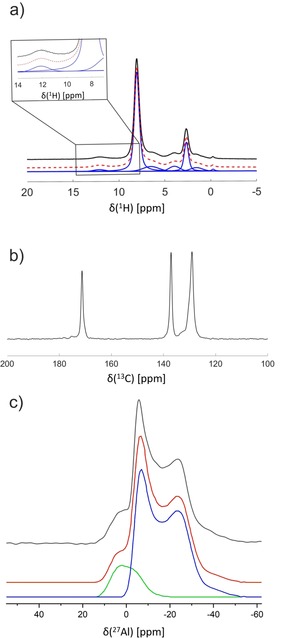
a) ^1^H SP, b) ^13^C CP, and c) ^27^Al SP MAS NMR spectra of Al‐MIL‐53. The ^1^H SP NMR spectrum (black lines) is presented together with a deconvolution by using pseudo‐Voigt profiles (red and blue lines). The experimental ^27^Al SP NMR spectrum (black) was deconvoluted with two resonances (blue and green) with line shapes typical for a second‐order quadrupolar broadening. The resulting simulated line shape is depicted as a red line.

In the ^1^H single‐pulse (SP) NMR spectrum (Figure 4 a) two dominant features at 8.0 and 2.6 ppm with an intensity ratio of 4:1 are discernable, which were attributed to the four aromatic protons of the terephthalate linkers and the single proton of the bridging hydroxide groups (Al‐OH‐Al) between two AlO_6_ octahedra.^11^, ^30^

The small low‐field resonance at 13 ppm is characteristic for unconnected carboxylic acid groups of the linkers. Because the presence of free terephthalic acid is unlikely due to the synthesis and purification conditions, this signal implies that roughly 15 % of the linkers (Table S9) are in a defect state with only one of the two carboxylic acid groups being deprotonated and coordinated to the Al^3+^ ions. This is also in line with a weak intensity IR band at 1702 cm^−1^ that is ascribed to aromatic carboxylic acid groups (Figure S23, Table S7). The resonances at 6.3 and 2.6 ppm are attributed to residual acetamide arising from the decomposition of acetonitrile with approximately one acetamide molecule per 10 linker molecules.^31^

A further resonance at 3.9 ppm is caused by adsorbed water molecules.^32^ This assignment is in line with the spectral fingerprint of the ^13^C CP MAS (cross‐polarization magic‐angle‐spinning) spectrum (Figure 4 b). The resonances at 130 and 137 ppm are associated with the aromatic CH and quaternary carbon atoms of the linker. The resonance at 171 ppm is assigned to carboxylate groups of connecting terephthalate linkers. Again, its downfield shoulder corroborates the existence of small residuals of protonated groups, as proposed above.^11^ Furthermore, a small resonance in the aliphatic region at 21 ppm (Figure S28) matches residual acetamide.

The ^27^Al single‐pulse magic‐angle‐spinning (SP MAS) NMR spectrum (Figure 4 c) reveals two distinctively different aluminum coordination environments. Both resonances exhibit the typical shapes for a second‐order quadrupolar broadening. The 2D satellite‐transition magic‐angle‐spinning (STMAS) spectrum (Figure S29) furthermore reveals a distribution of about 1 MHz for the quadrupolar coupling constants, probably arising from the disorder caused by the −COOH defects within the framework. The main component (blue line, Figure 4 c), with an isotropic chemical shift *δ*_iso_=2 ppm, a quadrupolar coupling constant of C_q_=8.8 MHz, and an anisotropy of η_q_=0 is typical for aluminum in a defect‐free Al‐MIL‐53 environment.^11^ The significant high‐field shift of *δ*_iso_ of about 9.6 ppm together with the reduction of C_q_ to 6.0 MHz for the minority contribution (green line, Figure 4 c) are suggestive of aluminum in a coordination with less than six equally strong contacts.^33^ The integrated intensity amounts to 16 % of the overall signal intensity, which matches the defect concentration as determined from the ^1^H MAS NMR spectra.

**Figure 5:**
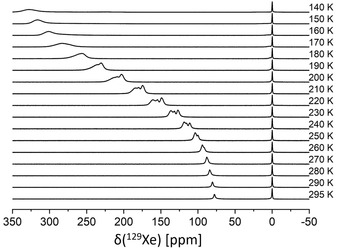
Single‐pulse NMR spectra of hyperpolarized ^129^Xe of Al‐MIL‐53 plotted as function of the temperature. Gas pressure was 5×10^5^ Pa with 5×10^3^ Pa xenon partial pressure.

To additionally probe the stability of the large‐pore form as a function of temperature under the influence of hydrophobic guest molecules, like xenon,^34^ variable‐temperature (vt) ^129^Xe NMR spectra (Figure 5) were recorded, using hyperpolarized xenon gas.^35^ At RT two resonances at 0 ppm typical for gas‐phase xenon and at 75 ppm characteristic for xenon adsorbed within the large‐pore form of Al‐MIL‐53 are visible.^34^, ^36^ Towards lower temperature the chemical shift for ^129^Xe adsorbed in Al‐MIL‐53 slowly and continuously increases towards 330 ppm at 140 K. The absence of a bistable state around 220 K signaled by a discontinuous increase of the shift by roughly 40 ppm^34^, 36a demonstrates that the present Al‐MIL‐53 sample does not change from a large‐ to a narrow‐pore form upon cooling. Between 260 to 190 K the signal is composed of several, overlapping shapes that, in accordance to the literature, can be assigned to clusters of adsorbed xenon atoms.^37^

This significant number of defects in the present Al‐MIL‐53 material is very likely to interfere with its lattice dynamics and might thus inhibit the typical breathing behavior of the framework as demonstrated by the water adsorption (Figure 3) and vt ^129^Xe NMR experiments (Figure 5).

**Figure 6:**
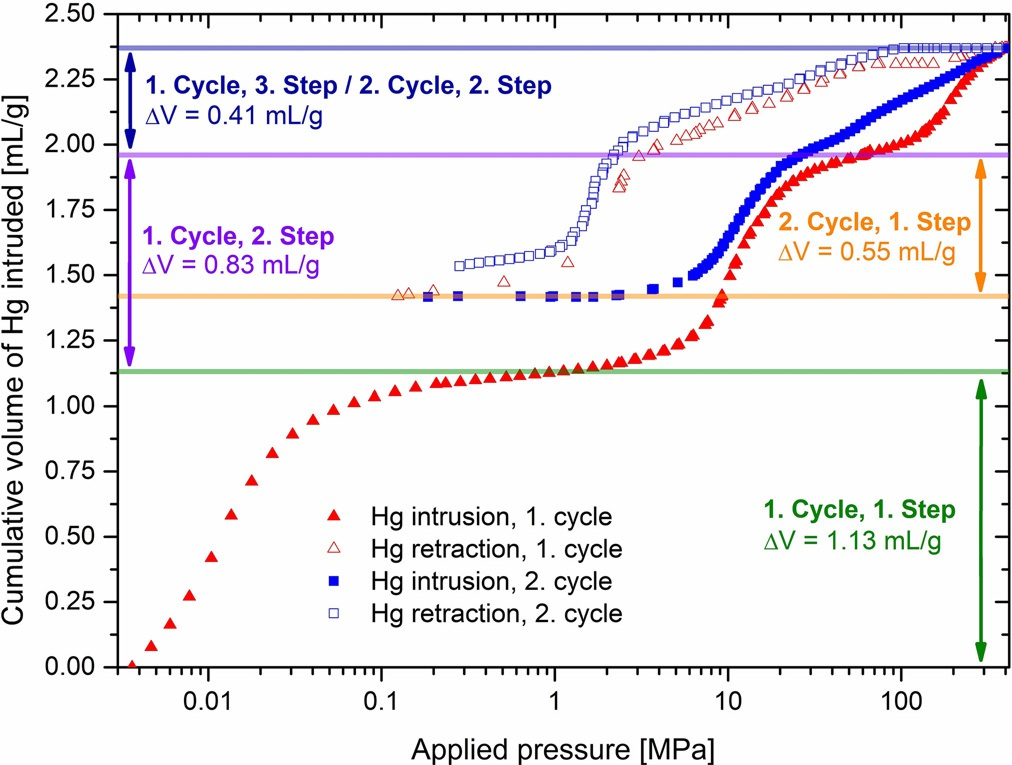
Cumulative volume of intruded mercury in two intrusion–extrusion cycles as a function of the applied pressure for Al‐MIL‐53.

Mercury intrusion porosimetry of Al‐MIL‐53 over two cycles revealed that the solid, in spite of showing no breathing effect upon water adsorption or cooling, can be forced to breathe reversibly by applying moderate pressures (≈60 MPa). The applied pressure is significantly higher than the pressure required to contract standard Al‐MIL‐53 (18 MPa), which shows an irreversible structural switching.20c Furthermore, the resulting compression‐decompression curves (Figure 6) exhibit a different evolution than those in previous studies dedicated to similar materials.20b–20e, 38

**Figure 7:**
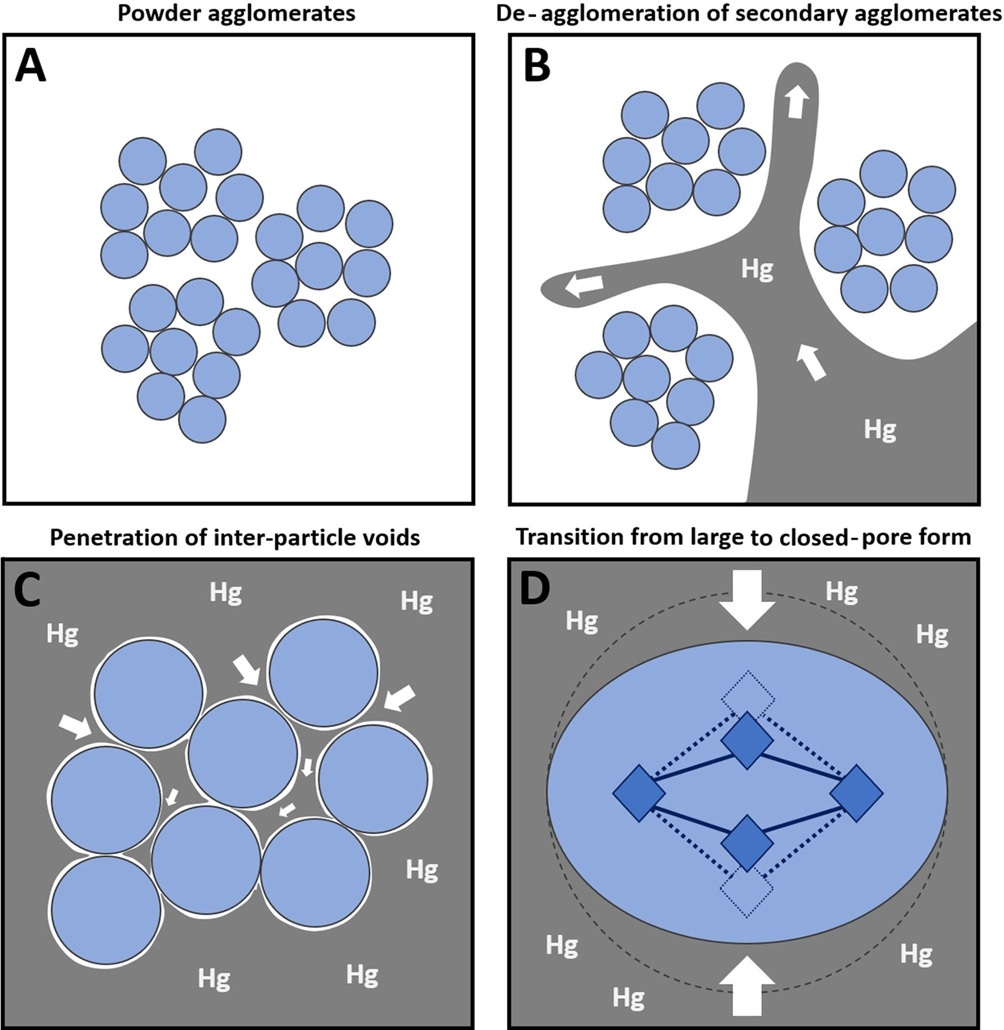
Schematic illustration of the mercury intrusion process for Al‐MIL‐53. A) Agglomerated powder particles (represented as blue spheres for simplification) before mercury intrusion. B) De‐agglomeration of secondary powder agglomerates. C) Mercury penetration of inter‐particle voids (primary agglomerates). D) Phase transition from large‐ to closed‐pore form. For the closed‐pore state blue filled diamonds represent the Al‐MIL‐53 chain‐type IBU viewed along the *c*‐axis and dark blue sticks represent the terephthalate linkers. In the same way dashed lines and nonfilled objects indicate the large‐pore state before pore closure.

The first step of the first cycle represents the filling of the intrusion cell and the de‐agglomeration of the powder particles (secondary agglomerates) (Figure 7 B). Because the powder is composed of very small intergrown crystallites (Figure S25–S27), the subsequent step can be assigned to the mercury penetration into the inter‐particle voids (primary agglomerates) (Figure 7 C). This is also in line with the average particle size of 140 to 270 nm as determined from mercury intrusion itself by the Mayer–Stowe method.^39^ The first two steps are indeed typical for aggregated powders that are not porous towards mercury.^39^, ^40^ The last step of the first cycle shows a volume variation (Δ*V*) of 0.41 mL g^−1^ which is similar to the material's micropore volume (0.46 mL g^−1^) as determined from nitrogen adsorption data (Table 1). It corresponds to the phase transition of Al‐MIL‐53 from its large‐pore form into its closed‐pore (cp) form because of the external compression by mercury (mercury cannot penetrate the small micropores of the framework) (Figure 7 D).

The calculated cell volume reduction of the large‐pore form based on the volume of mercury intruded is 566 Å^3^. Therefore, the obtained Al‐MIL‐53‐cp has a cell volume of 940 Å^3^, which is well in line with the value of 947 Å^3^ reported by Loiseau et al. for standard narrow‐pore Al‐MIL‐53.^11^ Upon retraction the structure opens again and the large‐pore form is again obtained (first retraction step). Afterwards, a major part but not all of the mercury is released from the inter‐particle voids (second retraction step). It is a well‐known phenomenon that mercury is not quantitatively expelled by such a sample because some mercury will be trapped in the larger inter‐particle pores.40b, 40c The second intrusion/retraction cycle further shows that the contraction of Al‐MIL‐53 is reversible.

Compared to the flexible version of the solid the contraction of the large to the closed form occurs at higher pressure (≈60 MPa vs. 18 MPa) with a similar variation of volume Δ*V*≈37 %.20c The associated work for one cycle of compression and decompression is close to 25 J g^−1^.

These findings are well in line with the presence of defects within the material as observed by NMR spectroscopy. Because of the defects, which are stabilizing the large‐pore form, more force is required to contract the framework. Nevertheless, the small size/degree of intergrowth of the Al‐MIL‐53 particles (Figure S25–S27) could be another factor that inhibits the breathing effect through mechanical hindrance.

## Conclusions

In conclusion, we were able to synthesize a number of different MOFs using acetonitrile as the solvent, demonstrating its potential in MOF syntheses. It is capable of sufficiently dissolving smaller organic linker molecules with low polarity at moderate temperatures while being less hazardous than many other aprotic polar solvents such as DMF. The three title compounds CAU‐10, Ce‐UiO‐66, and Al‐MIL‐53 were characterized in depth. The Ce‐MOF could be obtained in gram‐scale quantities for the first time because of increased redox stability in this solvent. Surprisingly, the choice of acetonitrile affected the breathing properties of the synthesized Al‐MIL‐53 drastically. While the typical structural flexibility upon water adsorption was absent, mercury intrusion measurements revealed that the material can be reversibly forced to breath when a pressure of about 60 MPa is applied, which is significantly higher than the pressure required to irreversibly contract standard Al‐MIL‐53 (18 MPa). This behavior can most likely be ascribed to defects within the structure of the material. These consist of linker units which only coordinate through one carboxylate group, whereas the second group remains protonated. 15 % of the linker molecules exhibit this defective state. Another factor could be the small size/degree of intergrowth of the particles that might lead to a mechanical inhibition of the breathing effect. The missing flexibility of Al‐MIL‐53 results in distinct water adsorption properties with a high uptake of 310 mg g^−1^ in the range between 0.45 and 0.65 p/p° making the material a potential candidate for indoor humidity control applications.

## Conflict of interest

The authors declare no conflict of interest.

## Supporting information

As a service to our authors and readers, this journal provides supporting information supplied by the authors. Such materials are peer reviewed and may be re‐organized for online delivery, but are not copy‐edited or typeset. Technical support issues arising from supporting information (other than missing files) should be addressed to the authors.

Supplementary
