# Supplementary material for: Solvent Impact on the Properties of Benchmark Metal–Organic Frameworks: Acetonitrile‐Based Synthesis of CAU‐10, Ce‐UiO‐66, and Al‐MIL‐53
Source: Chemistry. 2020 Mar 9;26(17):3877–83. doi: 10.1002/chem.201905376 (PMC7154691; doi:10.1002/chem.201905376)
Supplement: Supplementary file 1 — Supplementary [file CHEM-26-3877-s001.pdf]

CHEMISTRY
A European Journal

Supporting Information

Solvent Impact on the Properties of Benchmark Metal–Organic Frameworks: Acetonitrile-Based Synthesis of CAU-10, Ce-UiO-66, and Al-MIL-53

Sebastian Leubner,[a] Robert Stäglich,[b] Julia Franke,[a] Jannick Jacobsen,[a] Jonas Gosch,[a] Renée Siegel,[b] Helge Reinsch,[a] Guillaume Maurin,[c] Jürgen Senker,*[b] Pascal G. Yot,*[c] and Norbert Stock*[a]

chem_201905376_sm_miscellaneous_information.pdf

Supporting Information

Solvent Impact on the Framework Properties of Benchmark MOFs: Acetonitrile Based Synthesis of CAU-10, Ce-UiO-66 and Al-MIL-53

Sebastian Leubner [a], Robert Stäglich [b], Julia Franke [a], Jannick Jacobsen [a], Jonas Gosch [a], Renée Siegel [b], Helge Reinsch [a], Guillaume Maurin [c], Jürgen Senker*[b], Pascal G. Yot*[c] and Norbert Stock*[a]

Experimental Procedures 2

Chemicals 2

Instruments 2

Synthetic Procedures 4

Results and Discussion 7

Overview of syntheses and obtained products 7

Powder X-Ray Diffraction (PXRD) 8

Thermogravimetric & Elemental Analysis 14

Variable Temperature (VT) PXRD 17

Nitrogen & Water Adsorption 19

Infrared Spectroscopy 23

Scanning (SEM) & Transmission Electron Microscopy (TEM) 27

Solid-State NMR Spectroscopy 29

References 31

[a] MSc. S. Leubner, MSc. J. Franke, MSc. J. Jacobsen, MSc. J. Gosch, Dr. H. Reinsch, Prof. Dr. N. Stock
Department for Inorganic Chemistry
University of Kiel
Max-Eyth Straße 2, 24118 Kiel, Germany.
E-mail: Stock@ac.uni-kiel.de

[b] MSc. R. Stäglich, Dr. R. Siegel, Prof. Dr. J. Senker
Inorganic Chemistry III
University of Bayreuth
Universitätsstraße 30, 95447 Bayreuth, Germany.

[c] Dr. P. G. Yot, Prof. Dr. G. Maurin
Institut Charles Gerhard Montpellier (ICGM) UMR 5253
Université de Montpellier
CNRS ENSCM, CC 1505, Place Eugène Bataillon, F-43095
Montpellier cedex 05.

Experimental Procedures

Chemicals

All chemicals were purchased from ABCR, Alfa Aesar, Fluka, Grüssing, Sigma Aldrich, Honeywell Fluka, Merck or Walter CMP and used without further purification.

Table S1. Chemicals, corresponding CAS numbers, purities and suppliers.

Chemical	CAS	Purity	Supplier
Acetone	67-64-1	Grade: Pure	Walter CMP
Acetonitrile	75-05-8	99.9 %	Walter CMP
Aluminum nitrate nonahydrate	7784-27-2	> 98 %	Fluka
4,4'-biphenyldicarboxylic acid	787-70-2	97 %	ABCR
Cerium ammonium nitrate	16774-21-3	98 %	Alfa Aesar
Copper nitrate trihydrate	10031-43-3	99 %	Honeywell Fluka
N,*N*-dimethylformamide	68-12-2	99 %	Grüssing
Ethanol	64-17-5	99 %	Walter CMP
Fumaric acid	110-17-8	99 %	ABCR
Isophthalic acid	121-91-5	99 %	Sigma Aldrich
2,6-naphthalenedicarboxylic acid	1141-38-4	98 %	ABCR
Scandium nitrate pentahydrate	13465-60-6	99.9 %	ABCR
Terephthalic acid	100-21-0	98 %	ABCR
Triethylamine	121-44-8	> 99.5 %	Sigma Aldrich
Trimesic acid	554-95-0	95 %	Sigma Aldrich
Zinc nitrate hexahydrate	10196-18-6	> 99 %	Merck
Zinc acetate dihydrate	5970-45-6	99 %	Grüssing
Zirconium oxychloride octahydrate	13520-92-8	98 %	Sigma Aldrich
Zirconium tetrachloride	10026-11-6	98 %	ABCR

Instruments

Experimental details: Syntheses were carried out with PTFE lined steel autoclaves (max. volume 30 mL), Duran vials (max. volume 5 mL) or microwave glass vials (max. volume 4 mL) under solvothermal reaction conditions. Pressure-free synthesis were carried out with 250 or 500 mL round bottom flaks equipped with reflux condensers.

Powder X-ray diffraction (PXRD): Powder diffraction was performed with a STOE Stadi P Combi diffractometer (λ = 1.5406 Å), equipped with a MYTHEN detector. For structure determination EXPO2009, TOPAS Academics (v4) and Materials Studio 5.0 were used.

Variable temperature (VT) PXRD: PXRD measurements at different temperatures were carried out with a Stoe capillary furnace in 0.5 mm quartz capillaries.

Adsorption measurements: Nitrogen and water adsorption measurements were performed with a BELsorp max from BEL Japan Inc. at -196 °C for nitrogen and 25 °C for water. All samples were activated at elevated temperatures and under reduced pressure (10^{-2} kPa).

Thermogravimetric analysis: Thermogravimetric experiments were carried out on a NETSCH STA 429 CD analyzer using a heating rate of 4 K/min under flowing air with a flow rate of 75 mL/min.

Elemental analysis: Elemental analyses were performed with a HEKAtech Euro Elemental Analyzer set up for the determination of carbon, hydrogen, nitrogen and sulfur.

Infrared spectroscopy: IR spectra were collected with Bruker ALPHA-P A220/D-01 FTIR spectrometer equipped with an ATR (Attenuated Total Reflection) unit over a spectral range of 4000 to 400 cm^{-1}.

Scanning electron microscopy: SEM images were recorded with a Zeiss Gemini Ultra 55 Plus equipped with a silicon drift detector from Oxford.

Transmission electron microscopy: Bright-field images were collected on a JEOL JEM 2100 LaB_6 transmission electron microscope operating at 200 kV using a Gatan Orius SC200D CCD camera.

Mercury intrusion porosimetry: Mercury intrusion has been successfully used to provoke the contraction of porous solids like metal organic frameworks. [1-8] Powder of Al-MIL-53 was activated at 210 °C for 8 h under secondary vacuum prior to the measurement. The so obtained powder was then loaded into a powder penetrometer of 3.1126 mL volume with a stem volume of 0.4120 mL under argon atmosphere (H_2O < 5 ppm) using a glove box (Jacomex P-BOX). The mercury intrusion experiment was carried out within a pressure range of 0.003 to 413 MPa using a Micromeritics AutoPore IV 9500. Preliminary to the experiment the powder was outgassed at ~6.5 Pa for 15 minutes. The collected volume of mercury intruded was corrected by a blank, which was recorded under the same conditions of temperature and pressure using the same penetrometer, to obtain the absolute contracted volume as a function of the pressure.

Solid-state NMR spectroscopy: Solid-state NMR studies of ^{1}H (ν_0 = 600 MHz) and ^{27}Al (ν_0 = 156.34 MHz) were performed on a Bruker AvanceIII HD spectrometer with an external magnetic field of 14.1 T. The measurements were carried out in a 1.3 mm HFXY MAS triple-resonance wide bore probe with commercial ZrO_2 rotors. For the acquisition of ^{1}H single pulse (SP) NMR spectra, a RF nutation frequency of 200 kHz with a pulse length of 1.2 µs, 16 scans and a recycle delay of 5 s was set. ^{27}Al spectra were acquired after single pulse excitation with a RF field strength of ν_{nut} = 18 kHz, a pulse length of 1 µs, 64k scans and a recycle delay of 0.25 s. NMR parameters of ^{27}Al were determined by simulation of the experimental spectra with the QUADFIT program,[9] which accounts for effects of local disorder of the aluminum environment in the framework. For both the ^{1}H and the ^{27}Al NMR SP spectra the spinning speed was adjusted to 62.5 kHz.

^{13}C CPMAS NMR spectra were recorded on a Bruker AvanceIII HD spectrometer with an external magnetic field of 9.4 T and a ^{13}C Larmor frequency of 100.58 MHz. Acquisitions were performed setting a spinning speed of 9 and 12.5 kHz, respectively. For the measurements nutation frequencies of 100 kHz / 105 kHz were used, which amounts to a pulse length of 2.5 µs / 2.4 µs for the initial $\pi/2$ pulse. The contact times and nutation frequencies of the ^{13}C channel were adjusted to 3 ms and 62.5 kHz, respectively. For the ^{1}H channel the field strength was ramped linearly from 45 to 70 kHz / 50 to 75 kHz.[10] Heteronuclear decoupling was performed with a SPINAL-64 sequence[11] and a ^{1}H nutation frequency of 65 kHz / 80 kHz with phase increments of 5 degrees and pulse lengths of 5.9 / 6.9 µs. A recycle delay of 2 s was used with 106048 / 1200 repetitions, while 30 Hz line broadening

was applied prior the processing. All ^{1}H and ^{13}C spectra are referenced indirectly with respect to tetramethylsilane (TMS) using adamantane as secondary reference and the ^{27}Al NMR spectra were referenced using an aqueous solution of $Al(NO_3)_3$ adjusted to a pH of 0. The 2D ^{27}Al STMAS spectrum was recorded using the DQF-STMAS-split-t1 pulse sequence with an extended 768-step phase cycle. For suppression of CT-CT transitions a combination with echo / antiecho scheme was utilized in the indirect dimension.[12,13]

Variable temperature measurements of hyperpolarized ^{129}Xe spectra were carried out on a Bruker AvanceII NMR spectrometer at an external magnetic field of 7.05 T and a ν_0 of 83.43 MHz for the ^{129}Xe resonance. Hyperpolarized Xenon was supplied by our homebuilt polarizer, with a gas composition of 1 % v/v Xe and natural abundance of the ^{129}Xe isotope, 3 % v/v N_2 and Helium as buffer gas at a system pressure of $5\cdot10^5$ Pa. ^{129}Xe wideline SP NMR spectra were recorded in steps of 10 K, while new temperatures were regulated over a ramp (2 K/min) and an equilibration time of 5 minutes before starting the acquisition. The pulse length for the $\pi/2$ pulse was set to 3.3 µs at a RF nutation frequency of approximately 75 kHz. A recycle delay of 5 s was used to acquire a total of 32 scans for each temperature. The chemical shift was referenced to gaseous xenon extrapolated to zero pressure (0 ppm).

Synthetic Procedures

Synthesis of CAU-10: 2.69 g (7.18 mmol) aluminum nitrate nonahydrate, 1.19 g (7.18 mmol) isophthalic acid and 20 mL acetonitrile were placed in a PTFE lined steel autoclave. The autoclave was sealed and the mixture was heated to 130 °C in one hour. Then the temperature was kept for 22 hours. After the reaction mixture cooled down to room temperature, 2.53 g of a slightly yellowish solid were obtained via filtration.

Synthesis of Al-MIL-53: 1.44 g (3.83 mmol) coarsely ground aluminum nitrate nonahydrate, 0.56 g (3.35 mmol) terephthalic acid and 20 mL acetonitrile were placed in a PTFE lined steel autoclave. The autoclave was sealed and the mixture was heated to 130 °C in one hour. Then the temperature was kept for 22 hours. After the reaction mixture cooled down to room temperature, 0.67 g of a white solid were obtained via filtration and subsequent washing with 200 mL ethanol.

Synthesis of Ce-UiO-66: 5.32 g (32.02 mmol) terephthalic acid and 180 mL of acetonitrile were placed in a 500 mL round bottom flask and refluxed (oil bath temperature: 100 °C) under stirring (600 rpm). Afterwards 60 mL (32.02 mmol, 0.533 mol/L) of an aqueous solution of cerium ammonium nitrate ($(NH_4)_2[Ce(NO_3)_6]$) were added and the mixture was refluxed for another 2 hours. After the reaction mixture had cooled down to room temperature, 7.21 g of an orange-yellow precipitate were isolated via centrifugation.

Synthesis of standard Ce-UiO-66: Standard Ce-UiO-66 was prepared as described elsewhere.[14] 35.4 mg (0.213 mmol) terephthalic acid, 1.2 mL of DMF and a magnetic stirring bar were placed in a 8 mL Duran glass tube. Afterwards 0.4 mL (0.213 mmol, 0.533 mol/L) of an aqueous solution of cerium ammonium nitrate ($(NH_4)_2[Ce(NO_3)_6]$) were added and the Duran glass tube was sealed. Under stirring the mixture was heated for 15 minutes at 100 °C. After the reaction the precipitate was isolated by centrifuging the mixture and decanting off the mother liquor. The precipitate was redispersed in 2 mL DMF and centrifuged again. This process was repeated once before the solid was washed and centrifuged with acetone (2 mL) four times. Subsequent drying at 70 °C in air yielded 38.4 mg of a yellow solid.

Purification of CAU-10: For purification (removal of impurities) 1 g of crude product was stirred (400 rpm) in 50 mL of deionized water for 3 hours at room temperature, yielding 0.58 g of a white solid after filtration.

Purification of Al-MIL-53: For purification (removal of impurities) 0.67 g of crude product were stirred (600 rpm) in 20 mL of deionized water for 24 hours at room temperature, yielding 0.64 g of a white solid after filtration.

Purification of Ce-UiO-66: For purification (removal of impurities) 7.21 g of crude product were dispersed in 20 mL acetone and centrifuged. This procedure was repeated one more time before the product was stirred in 250 mL DMF for 24 hours (subsequent centrifugation) and thereafter in 250 ml acetone for another 24 hours. Finally, 4.89 g of an orange-yellow solid were obtained after centrifugation and drying at 70 °C in air.

Syntheses of additional MOFs/products

Synthesis of Zr-UiO-66: 96.6 mg (0.300 mmol) zirconium oxychloride octahydrate, 49.8 mg (0.300 mmol) terephthalic acid, 3 mL acetonitrile and a stirring bar were placed in a 5 mL Duran glass vial. The vial was sealed with a plastic cap and placed in an aluminum heating block. Subsequently, the mixture was heated at 92 °C heating block temperature for 5 hours under stirring (600 rpm). After the reaction mixture cooled down to room temperature, 98.6 mg of a white solid were obtained via filtration.

Synthesis of MOF-801: 1.61 g (5.000 mmol) zirconium oxychloride octahydrate, 0.58 g (5.000 mmol) fumaric acid and 50 mL of acetonitrile were placed in a 250 mL round bottom flask and refluxed for 17 hours (oil bath temperature: 100 °C) under stirring (1000 rpm). After the reaction mixture had cooled down to room temperature, 1.01 g of a white solid were obtained via filtration.

Synthesis of MIL-140A: 1.17 g (5.000 mmol) zirconium tetrachloride, 0.83 g (5.000 mmol) terephthalic acid and 50 mL of acetonitrile were placed in a 250 mL round bottom flask and refluxed for 20 hours (oil bath temperature: 100 °C) under stirring (800 rpm). After the reaction mixture had cooled down to room temperature, 1.32 g of a white solid were obtained via filtration.

Synthesis of HKUST-1: 222.1 mg (0.919 mmol) copper nitrate trihydrate, 127.5 mg (0.607 mmol) trimesic acid and 20 mL acetonitrile were placed in a PTFE lined steel autoclave. The autoclave was sealed and the mixture was heated to 130 °C in one hour. Then the temperature was kept for 22 hours. After the reaction mixture cooled down to room temperature, 234.3 mg of a light blue solid were obtained via filtration.

Synthesis of scandium terephthalate ($Sc_3(BDC)_2$): 409.8 mg (1.276 mmol) scandium nitrate pentahydrate, 212.1 mg (1.276 mmol) terephthalic acid and 20 mL acetonitrile were placed in a PTFE lined steel autoclave. The autoclave was sealed and the mixture was heated to 130 °C in one hour. Then the temperature was kept for 22 hours. After the reaction mixture cooled down to room temperature, 291.7 mg of a light yellow solid were obtained via filtration.

Synthesis comprising copper nitrate trihydrate and terephthalic acid: 244.0 mg (1.010 mmol) copper nitrate trihydrate, 167.8 mg (1.010 mmol) terephthalic acid and 20 mL acetonitrile were placed in a PTFE lined steel autoclave. The autoclave was sealed and the mixture was heated to 130 °C in one hour. Then the temperature was kept for 22 hours. After the reaction mixture cooled down to room temperature, 237.1 mg of a dark green/brown solid were obtained via filtration. The solid was transferred into a 20 ml glass vial and 18 mL of DMF as well as a stirring bar were added. After stirring the mixture for 30 min. it was filtrated and the solids were washed with 50 mL of acetone, yielding 183.7 mg of a dark green solid.

General procedure for syntheses comprising zinc salts as the metal source: 601.0 mg (2.020 mmol) zinc nitrate hexahydrate or 443.4 mg (2.020 mmol) zinc acetate dihydrate, 167.8 mg (1.010 mmol) terephthalic acid and 20 mL acetonitrile were placed in a PTFE lined steel autoclave. Optionally, 0.33 mL (2.424 mmol) of triethylamine could be added. The autoclave was sealed and the mixture was heated to 130 °C in one hour. Then the temperature was kept for 22 hours. After the reaction mixture cooled down to room temperature, 180-420 mg of solid were obtained via filtration. The solid was transferred into a 20 ml glass vial and 18 mL of DMF as well as a stirring bar were added. After stirring the mixture for 30 min. it was filtrated and the solids were washed with 50 mL of acetone, yielding 85-315 mg solid.

Synthesis comprising cerium ammonium nitrate and isophthalic acid: 58.5 mg (0.106 mmol) cerium ammonium nitrate ($(NH_4)_2[Ce(NO_3)_6]$), 26.6 mg (0.160 mmol) isophthalic acid and 0.8 mL acetonitrile as well as a stirring bar were placed in a 4 mL microwave glass vial and the vial was subsequently sealed with a plastic cap. The reaction mixture was heated at 150 °C for 4 hours under stirring (400 rpm) with a microwave oven. After the reaction mixture cooled down to room temperature, 26.2 mg of a white solid were obtained via filtration.

Syntheses comprising zirconium oxychloride octahydrate and 4,4'biphenyldicarboxylic or 2,6-naphthalene dicarboxylic acid: 1.61 g (5.000 mmol) zirconium oxychloride octahydrate and 1.21 g (5.000 mmol) 4,4'-biphenyldicarboxylic acid or 1.08 g (5.000 mmol) 2,6-naphthalenedicarboxylic acid as well as 50 mL of acetonitrile were placed in a 250 mL round bottom flask and refluxed for 20 hours (oil bath temperature: 100 °C) under stirring (1000 rpm). After the reaction mixture had cooled down to room temperature, 1.6-2.0 g of a white solid were obtained via filtration.

Results and Discussion

Overview of syntheses and obtained products

In order to underline the viability of acetonitrile as a synthesis solvent for MOFs, a screening of different metal ions and linker molecules was carried out. The resulting products and the most important synthetic details are given in Table S2. CAU-10, Ce-UiO-66 and Al-MIL-53 were chosen for an in-depth characterization, while the remaining compounds were only investigated via PXRD. Upon reacting cerium ammonium nitrate with isophthalic acid or when larger linker molecules like 4,4'-biphenyldicarboxylic or 2,6-naphthalenedicarboxylic acid were utilized, mostly unreacted linker was reobtained. In the latter case insufficient dissolution of the large linker molecules in acetonitrile can explain the observation. The reactions using Zn^{2+} salts resulted in crystalline reaction products that could not be assigned to MOF-5, the expected reaction product in DMF as the solvent.

Table S2. Products and selected synthetic details for all syntheses carried out with acetonitrile as the solvent.

Product	Metal source (M)	Linker (L)	Additive	M : L ratio	Reaction vessel	Temperature	Duration
CAU-10	$Al(NO_3)_3{\cdot}9H_2O$	Isophthalic acid	-	1 : 1	Autoclave	130 °C	24 h
Al-MIL-53	$Al(NO_3)_3{\cdot}9H_2O$	Terephthalic acid	-	1 : 0.88	Autoclave	130 °C	24 h
Ce-UiO-66	$(NH_4)_2[Ce(NO_3)_6]$	Terephthalic acid	-	1 : 1	Round bottom flask	Reflux	2 h
Linker and X-ray amorphous solid	$(NH_4)_2[Ce(NO_3)_6]$	Isophthalic acid	-	1 : 1.5	Sealed microwave vial	150 °C	4 h
Zr-UiO-66	$ZrOCl_2{\cdot}8H_2O$	Terephthalic acid	-	1 : 1	Sealed Duran vial	92 °C	5 h
MOF-801	$ZrOCl_2{\cdot}8H_2O$	Fumaric acid	-	1 : 1	Round bottom flask	Reflux	17 h
Linker and X-ray amorphous solid	$ZrOCl_2{\cdot}8H_2O$	4,4'-biphenyl-dicarboxylic acid	-	1 : 1	Round bottom flask	Reflux	20 h
Linker and X-ray amorphous solid	$ZrOCl_2{\cdot}8H_2O$	2,6-naphthalene-dicarboxylic acid	-	1 : 1	Round bottom flask	Reflux	20 h
MIL-140A	$ZrCl_4$	Terephthalic acid	-	1 : 1	Round bottom flask	Reflux	20 h
HKUST-1	$Cu(NO_3)_2{\cdot}3H_2O$	Trimesic acid	-	1 : 0.66	Autoclave	130 °C	24 h
Not identified (**A**)	$Cu(NO_3)_2{\cdot}3H_2O$	Terephthalic acid	-	1 : 1	Autoclave	130 °C	24 h
$Sc_2(BDC)_3$	$Sc(NO_3)_3{\cdot}5H_2O$	Terephthalic acid	-	1 : 1	Autoclave	130 °C	24 h
Not identified (**B**)	$Zn(OAc)_2{\cdot}2H_2O$	Terephthalic acid	-	1 : 0.5	Autoclave	130 °C	24 h
Not identified Phase mixture (**C**)	$Zn(OAc)_2{\cdot}2H_2O$	Terephthalic acid	Et_3N	1 : 0.5	Autoclave	130 °C	24 h
Not identified (**D**)	$Zn(NO_3)_2{\cdot}6H_2O$	Terephthalic acid	-	1 : 0.5	Autoclave	130 °C	24 h
Not identified Phase mixture (**E**)	$Zn(NO_3)_2{\cdot}6H_2O$	Terephthalic acid	Et_3N	1 : 0.5	Autoclave	130 °C	24 h

Powder X-Ray Diffraction (PXRD)

Title compounds (CAU-10, Ce-UiO-66, Al-MIL-53)

In the following section the PXRD patterns of the as synthesized and purified title MOFs are compared to their theoretical patterns. Al-MIL-53 is also compared to the activated (open pore) form of V-MIL-47 [V(OH)(BDC)] to point out its similarity. V-MIL-47 is as well as Al-MIL-53 composed of trans corner sharing MO_6 octahedra. Furthermore, LEBAIL fits of Al-MIL-53, CAU-10 and Ce-UiO-66 are shown.

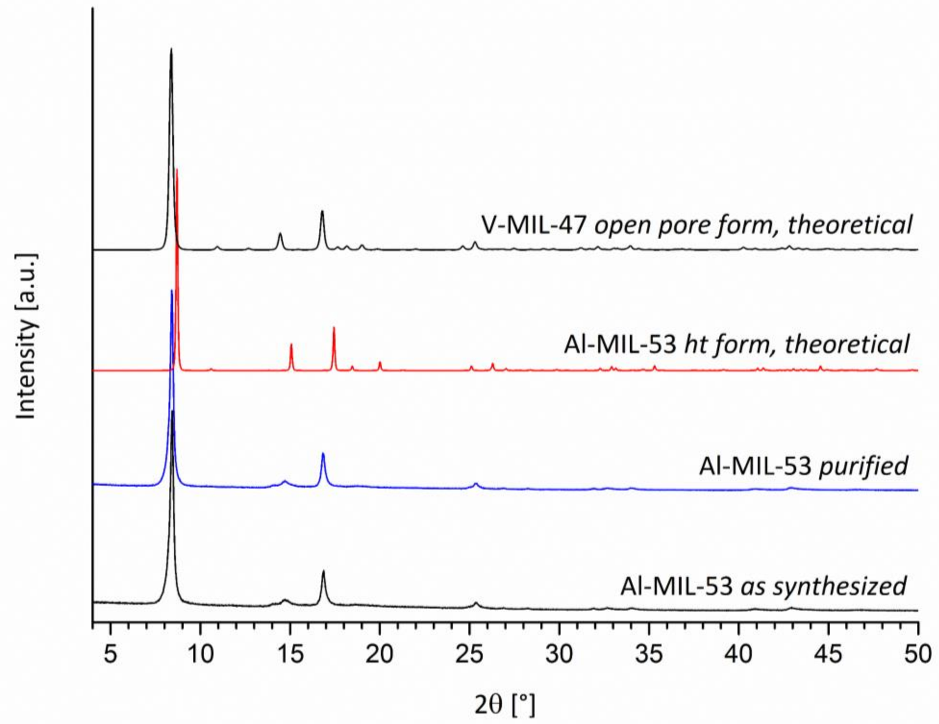

Figure S1. PXRD patterns of as synthesized and purified Al-MIL-53 in comparison with theoretical patterns of Al-MIL-53 (ht form)[15] and V-MIL-47 (open pore form).[16]

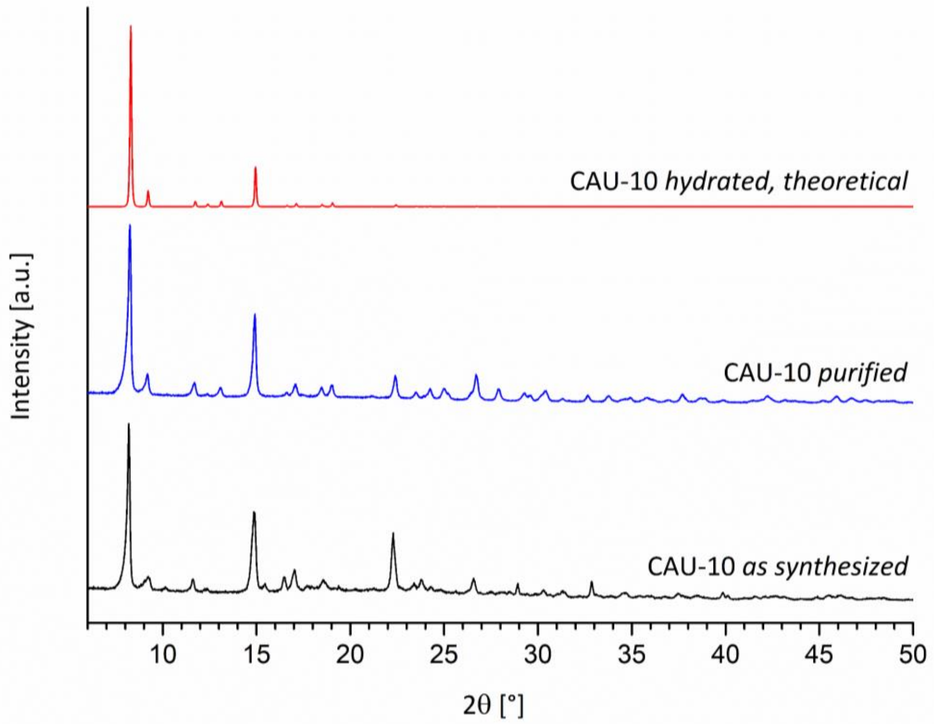

Figure S2. PXRD patterns of as synthesized and purified CAU-10 in comparison with its theoretical pattern.[17] The as synthesized compound shows no reflections of unreacted isophthalic acid but represents a mixture of hydrated and non-hydrated CAU-10.

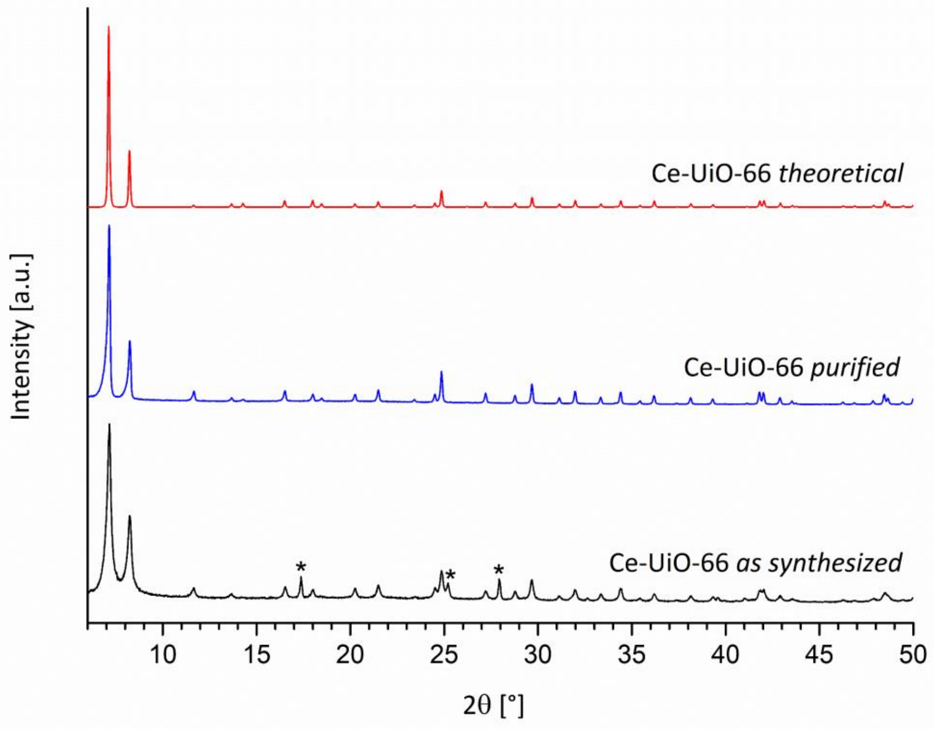

Figure S3. PXRD patterns of as synthesized and purified Ce-UiO-66 in comparison with its theoretical pattern.[14] Reflections of unreacted terephthalic acid are highlighted with asterisks.

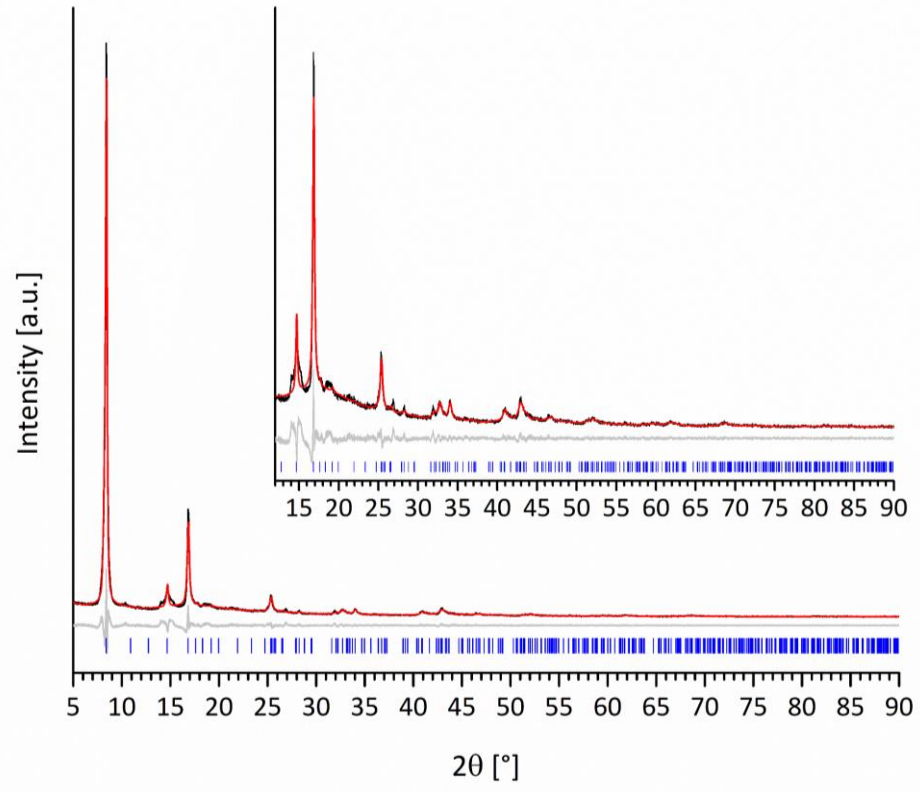

Figure S4. PXRD pattern of purified Al-MIL-53 (black) with LeBail fit (red), difference (grey) and allowed reflections (blue).

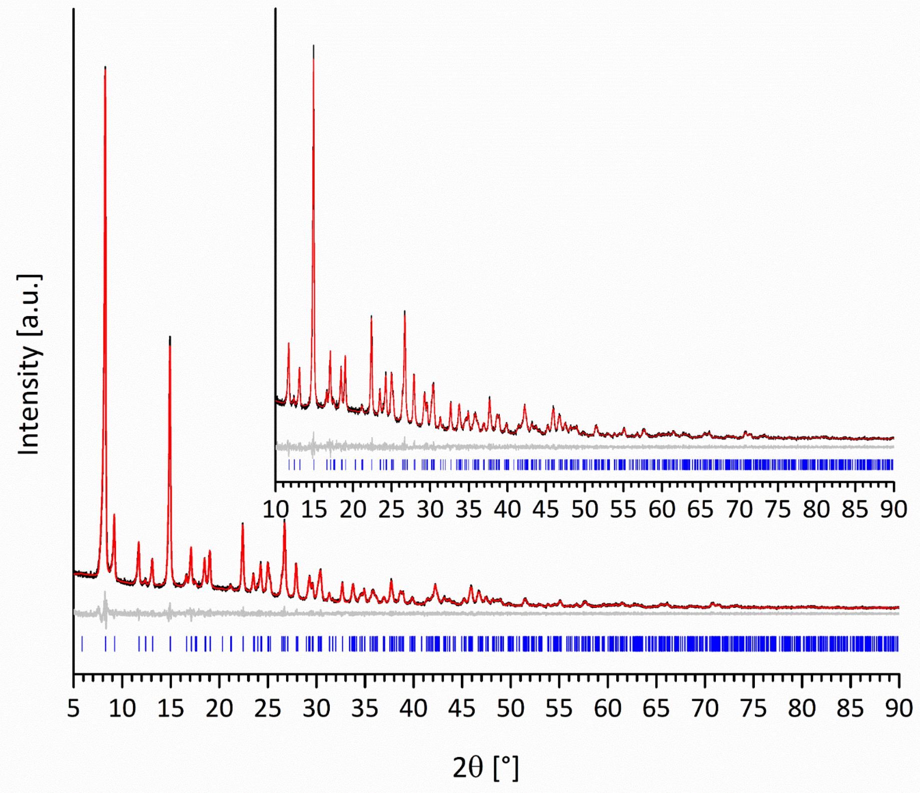

Figure S5. PXRD pattern of purified CAU-10 (black) with LeBail fit (red), difference (grey) and allowed reflections (blue).

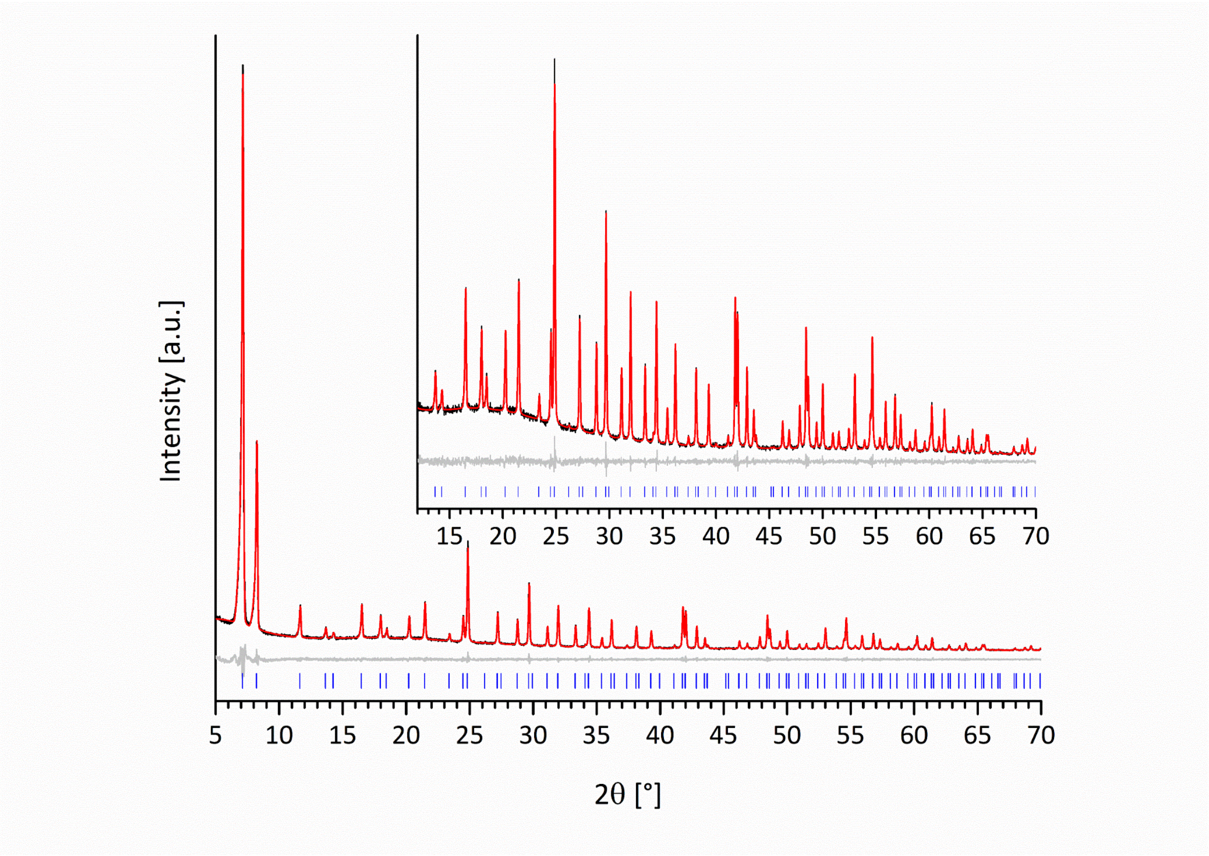

Figure S6. PXRD pattern of purified Ce-UiO-66 (black) with LeBail fit (red), difference (grey) and allowed reflections (blue).

Table S3. Unit cell parameters of CAU-10, Ce-UiO-66 and Al-MIL-53 in comparison with reported values.

Parameter	CAU-10 LeBail fit	CAU-10[17] (hydrated form)	Ce-UiO-66 LeBail fit	Ce-UiO-66[14]	Al-MIL-53 LeBail fit	V-MIL-47[16] (activated form)	Al-MIL-53[15] (ht form)
Crystal system	Tetragonal	Tetragonal	Cubic	Cubic	Orthorhombic	Orthorhombic	Orthorhombic
Space Group	$I4_1$	$I4_1$	$Fm\bar{3}m$	$Fm\bar{3}m$	$Imma$	$Pnma$	$Imma$
a [Å]	21.2874(5)	21.2928(4)	21.5073(1)	21.4727(3)	6.703(1)	6.8179(12)	6.6085(9)
b [Å]	21.2874(5)	21.2928(4)	21.5073(1)	21.4727(3)	16.209(4)	16.143(3)	16.675(3)
c [Å]	10.7143(3)	10.7304(3)	21.5073(1)	21.4727(3)	13.868(3)	13.939(2)	12.813(2)
α [°]	90.000	90.000	90.000	90.000	90.000	90.000	90.000
β [°]	90.000	90.000	90.000	90.000	90.000	90.000	90.000
γ [°]	90.000	90.000	90.000	90.000	90.000	90.000	90.000
Volume [Å^3]	4855.3(3)	4865.0(2)	9948.52(15)	9900.6(4)	1507.0(6)	1534.2(5)	1412.0(4)
r_{wp}	4.855	-	4.514	2.650	8.908	-	-
GOF	1.213	-	1.564	2.268	2.163	-	-

Other MOFs/products

The PXRD patterns of all other products are shown in Figure S7-9. MOF products are also compared to their theoretical pattern.

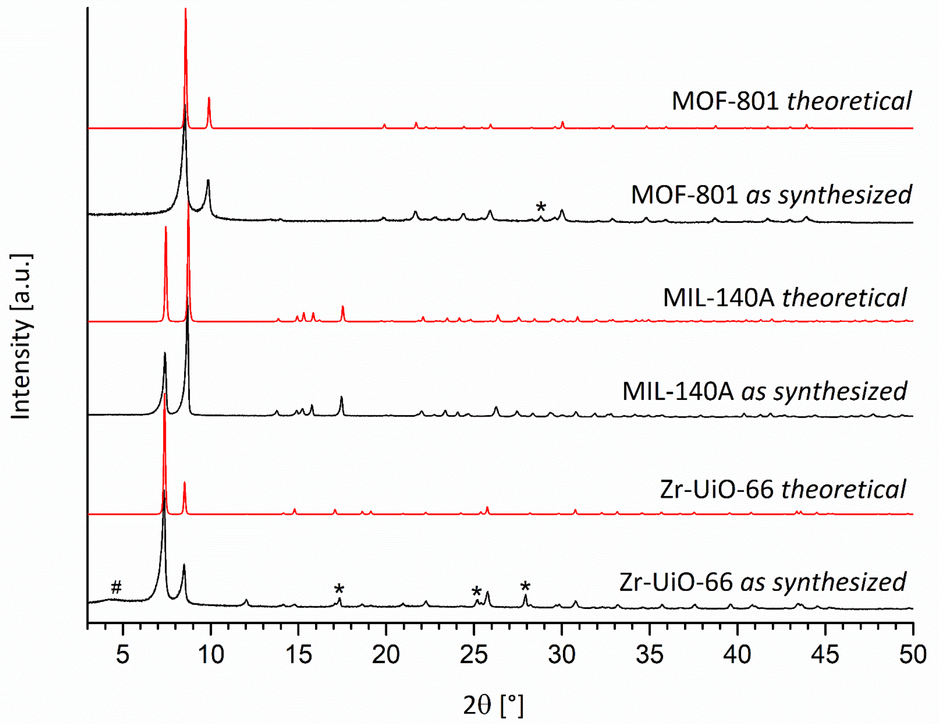

Figure S7. PXRD patterns of as synthesized Zr-UiO-66, MIL-140A and MOF-801 in comparison with their theoretical patterns.[18-20] Reflections of unreacted linker molecules (terephthalic acid for Zr-UiO-66 and fumaric acid for MOF-801) are highlighted with asterisks. Typical superstructure reflections for defective Zr-UiO-66 as described by Cliffe *et al.*[21] are marked with a hash symbol.

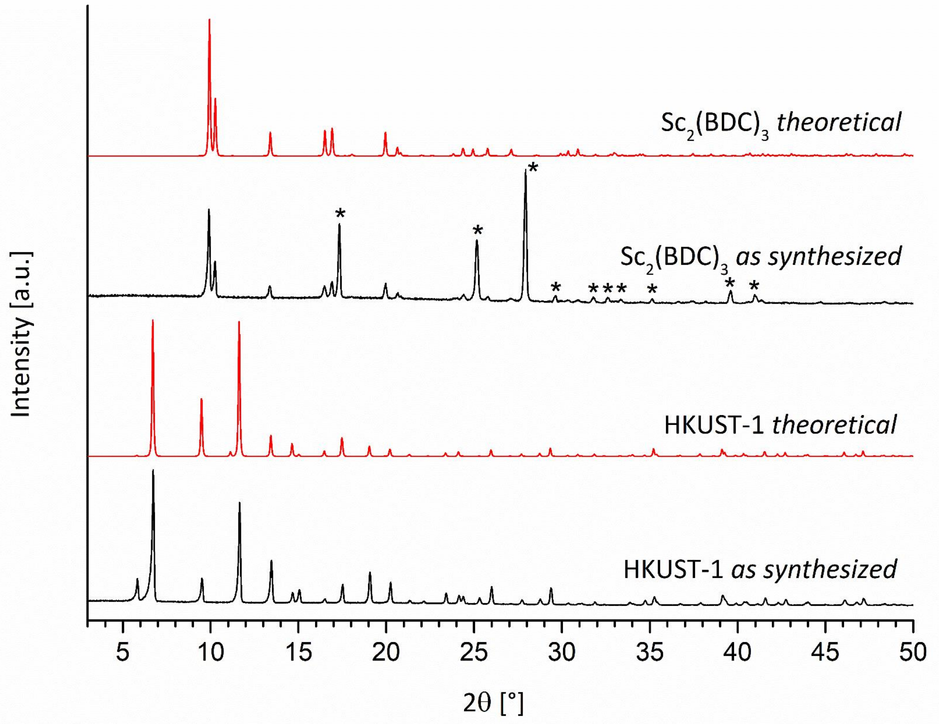

Figure S8. PXRD patterns of as synthesized HKUST-1 and scandium terephthalate ($Sc_2(BDC)_3$) in comparison with their theoretical patterns.[22,23] Reflections of unreacted terephthalic acid are highlighted with asterisks.

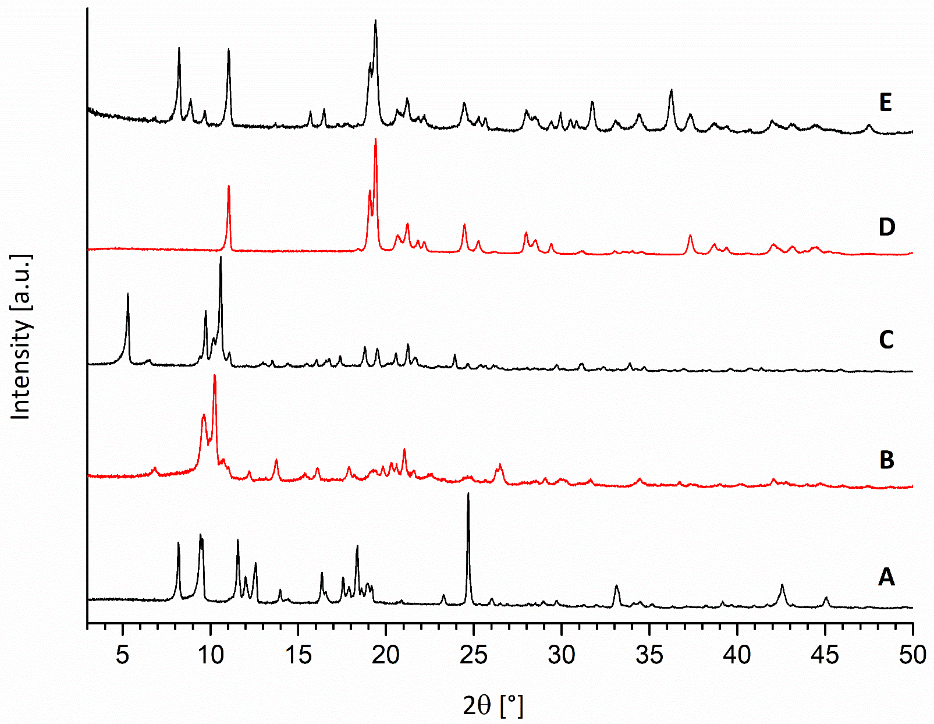

Figure S9. PXRD patterns of the non-identified compounds **A**-**E** (see Tab. S2). The PXRD patterns of **C** and **E** show that these products are phase mixtures, since reflections of **B** and **D** can be observed within them, respectively.

Thermogravimetric & Elemental Analysis

Thermogravimetric and elemental analyses were carried out for all purified compounds in order to confirm their compositions. Good matches of theoretical and experimental compositions were obtained for CAU-10 and Al-MIL-53. A defect concentration of 15 %, as determined via solid-state NMR spectroscopy, was considered for the theoretical composition of Al-MIL-53. The experimental data of Ce-UiO-66 could only be matched when 10 % cluster defects and large quantities of adsorbed solvent molecules (water, acetone) as well as acetamide were assumed. Nevertheless, the presence of the adsorbed species was confirmed via IR spectroscopy (section Infrared Spectroscopy). The number of adsorbed water molecules per formula unit (35) is also in the range the maximum water capacity (35.8 water molecules per formula unit) as determined via water adsorption (Fig. S20).

Table S4. Results of the thermogravimetric analyses for CAU-10, MIL-53 and Ce-UiO-66 including the TG step, the removed species, the temperature range as well as the measured and calculated wt% values.

Name	Sum formula	TG step	Removed species	Temperature range [°C]	wt% measured	wt% calculated
CAU-10	$[Al(OH)(ISO)]\cdot 3H_2O$	1	Water	< 50 - 110	21.30	20.62
		2	Linker	110 - 800	59.21	59.94
		Residue	Al_2O_3	800 °C	19.62	19.45
Al-MIL-53	$[Al(OH)_{1.15}(BDC)_{0.85}(BDC\text{-}H)_{0.15}]$ $\cdot 0.2H_2O\cdot 0.1(C_2H_5NO)$ (defect concentration determined via solid-state NMR spectroscopy)	1	Water	< 50 - 100	1.30	1.64
		2	Acetamide	100 - 350	2.90	2.68
		3	Linker	350 - 800	72.21	72.54
		Residue	Al_2O_3	800	23.59	23.14
Ce-UiO-66	$[Ce_{5.4}O_{3.6}(OH)_{3.6}(BDC)_{4.8}$ $(BDC\text{-}H)_{1.2}]\cdot 35H_2O\cdot 3(C_2H_5NO)$ $\cdot 4(C_3H_6O)$	1	Water/Acetone	< 50 - 120	30.33	29.74
		2	Acetamide	120 - 270	7.09	6.11
		3	Linker	270 - 800	31.40	32.12
		Residue	CeO_2	800	31.18	32.03

Table S5. Results of the elemental analyses for CAU-10, MIL-53 and Ce-UiO-66 including the elements and their measured and calculated %-values.

Name	Sum formula	Element	% measured	% calculated
CAU-10	$Al(OH)(ISO)\cdot 3H_2O$	Carbon	35.47	36.65
		Hydrogen	3.75	4.23
		Nitrogen	0.00	0.00
MIL-53	$[Al(OH)_{1.15}(BDC)_{0.85}(BDC\text{-}H)_{0.15}]$ $\cdot 0.2H_2O\cdot 0.1(C_2H_5NO)$	Carbon	45.02	44.70
		Hydrogen	2.72	2.84
		Nitrogen	0.32	0.64
Ce-UiO-66	$[Ce_{5.4}O_{3.6}(OH)_{3.6}(BDC)_{4.8}$ $(BDC\text{-}H)_{1.2}]\cdot 35H_2O\cdot 3(C_2H_5NO)$ $\cdot 4(C_3H_6O)$	Carbon	25.89	27.32
		Hydrogen	3.27	4.79
		Nitrogen	1.50	1.45

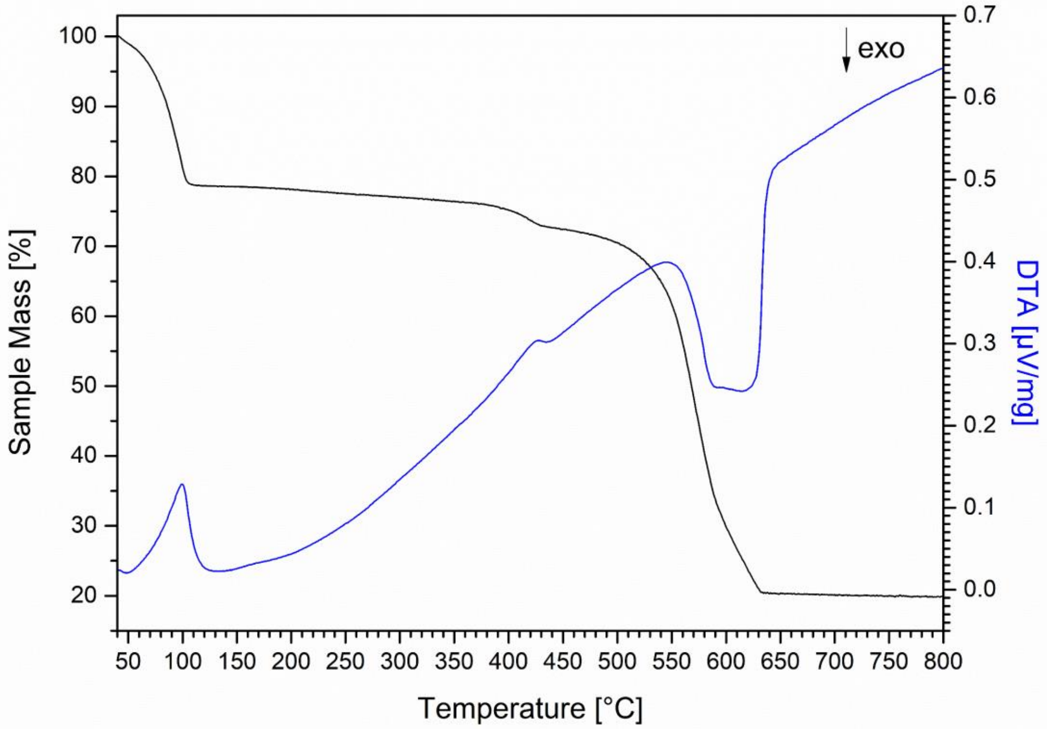

Figure S10. Combined TG/DTA data for CAU-10.

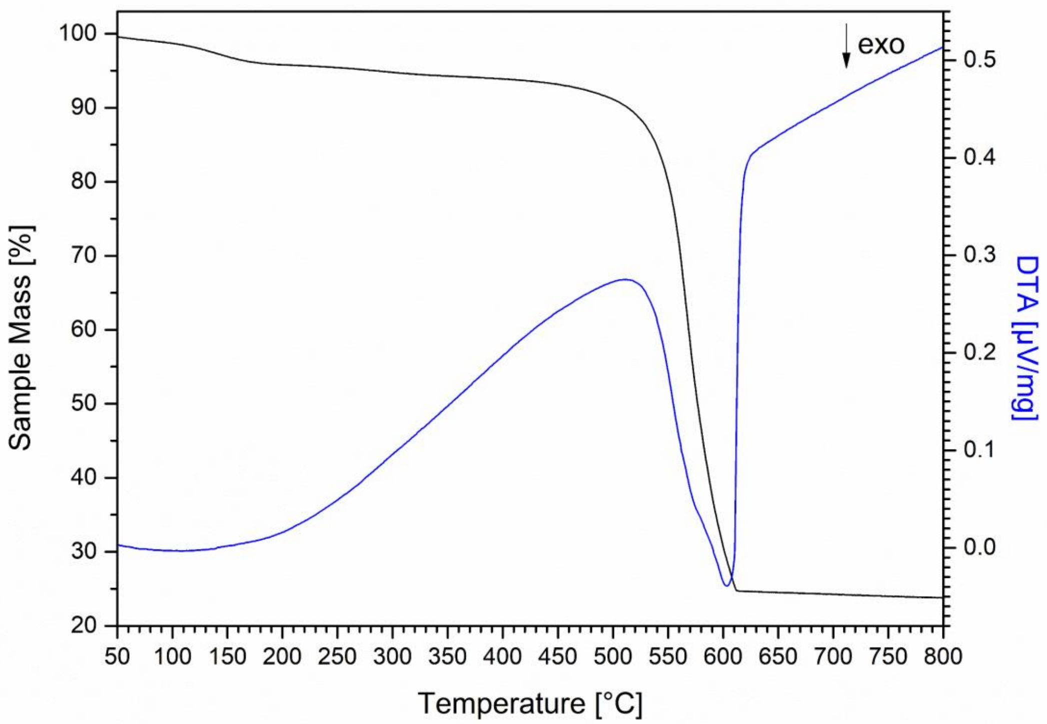

Figure S11. Combined TG/DTA data for Al-MIL-53.

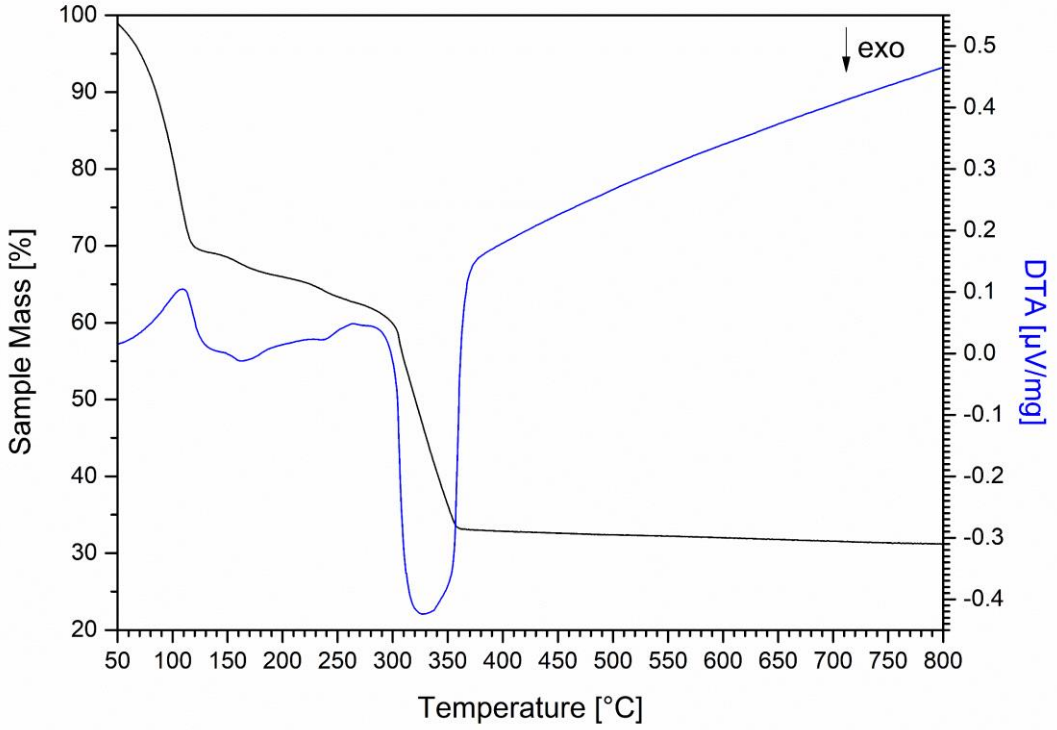

Figure S12. Combined TG/DTA data for Ce-UiO-66.

Variable Temperature (VT) PXRD

To investigate possible temperature-induced phase transitions X-ray powder diffraction measurements at different temperatures were carried out with purified Al-MIL-53 (see Synthetic Procedures). The sample was heated from 30 to 700 °C. Between 90 and 500 °C PXRD patterns were recorded in 10 °C steps. Within the remaining ranges (30 to 90 °C and 500 to 700 °C) 30 °C and 25 °C steps were applied, respectively. Every PXRD pattern was directly recorded after reaching the target temperature. The material shows a phase transition at ~500 °C into a low crystalline phase and decomposes right after at ~550 °C. It is very likely that the observed phase is a semi-amorphous decomposition intermediate.

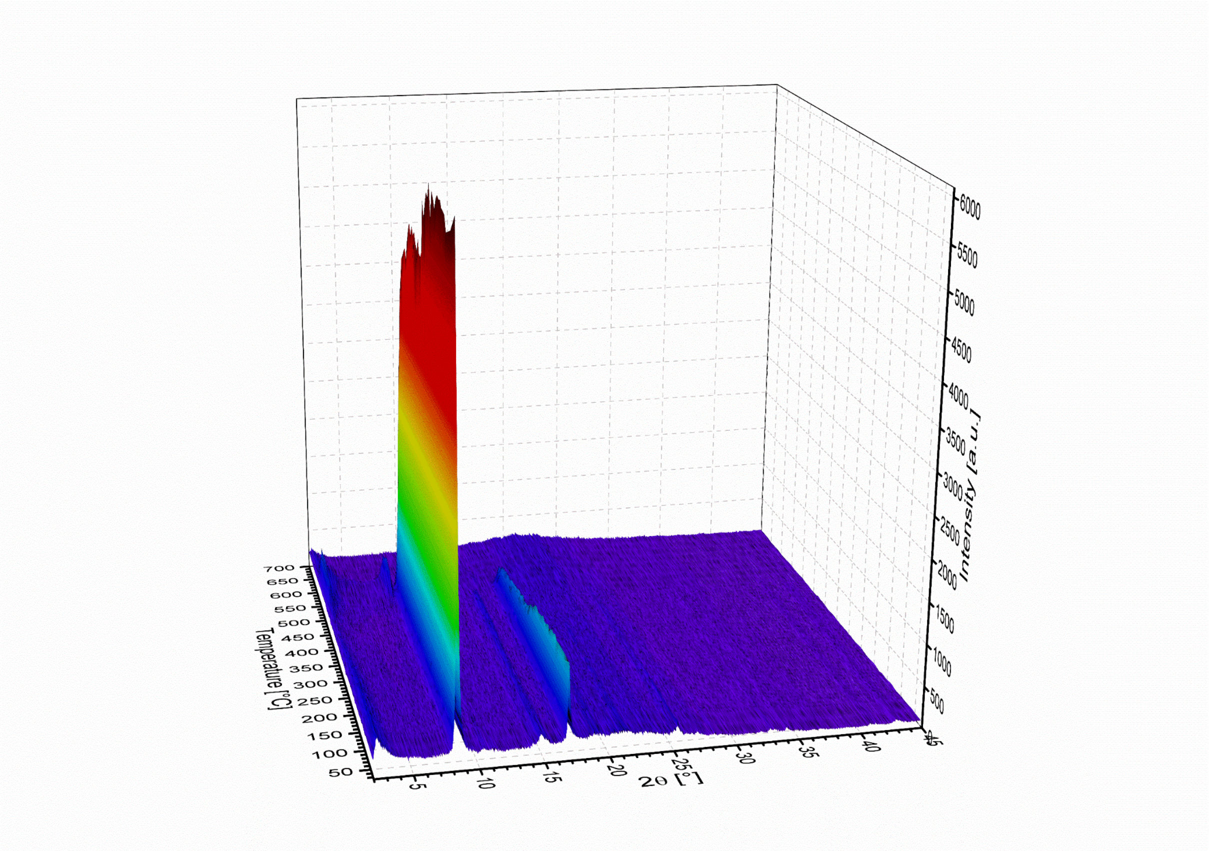

Figure S13. 3D representation of VT-PXRD data of Al-MIL-53.

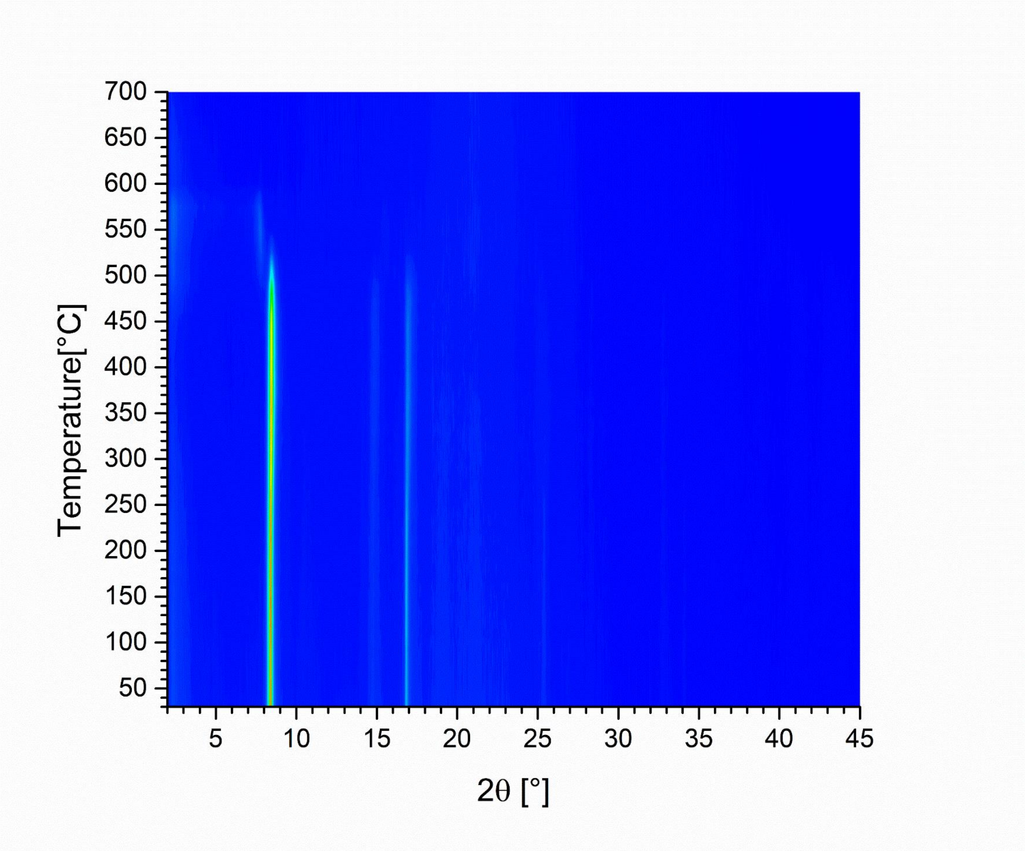

Figure S14. 2D representation of VT-PXRD data of Al-MIL-53.

Nitrogen & Water Adsorption

Nitrogen and water adsorption experiments were carried out in order to determine BET areas, micropore volumes and water adsorption characteristics. In case of nitrogen adsorption CAU-10, Al-MIL-53 and Ce-UiO-66 were also compared before and after their purification processes in order to determine the influence of impurities. To compare the water adsorption properties of Ce-UiO-66 from this work to standard Ce-UiO-66, the latter was prepared as described in the literature[14] (see Synthetic Procedures) and a water adsorption isotherm was recorded (Fig. S21).

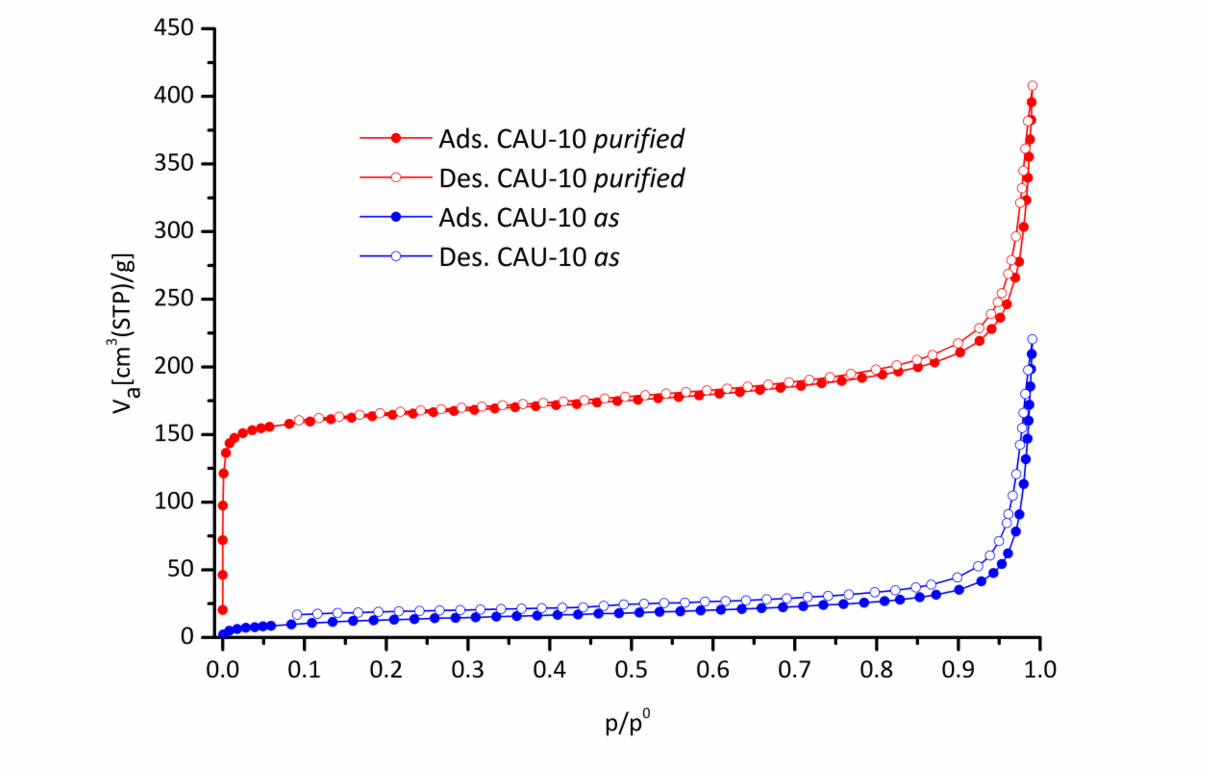

Figure S15. Nitrogen adsorption isotherm of CAU-10 (activation: 150 °C under reduced pressure, 2 h) before and after purification. The as synthesized compound shows nearly no nitrogen uptake due to impurities like acetamide or residual metal species which are blocking the pores/decreasing the specific surface area.

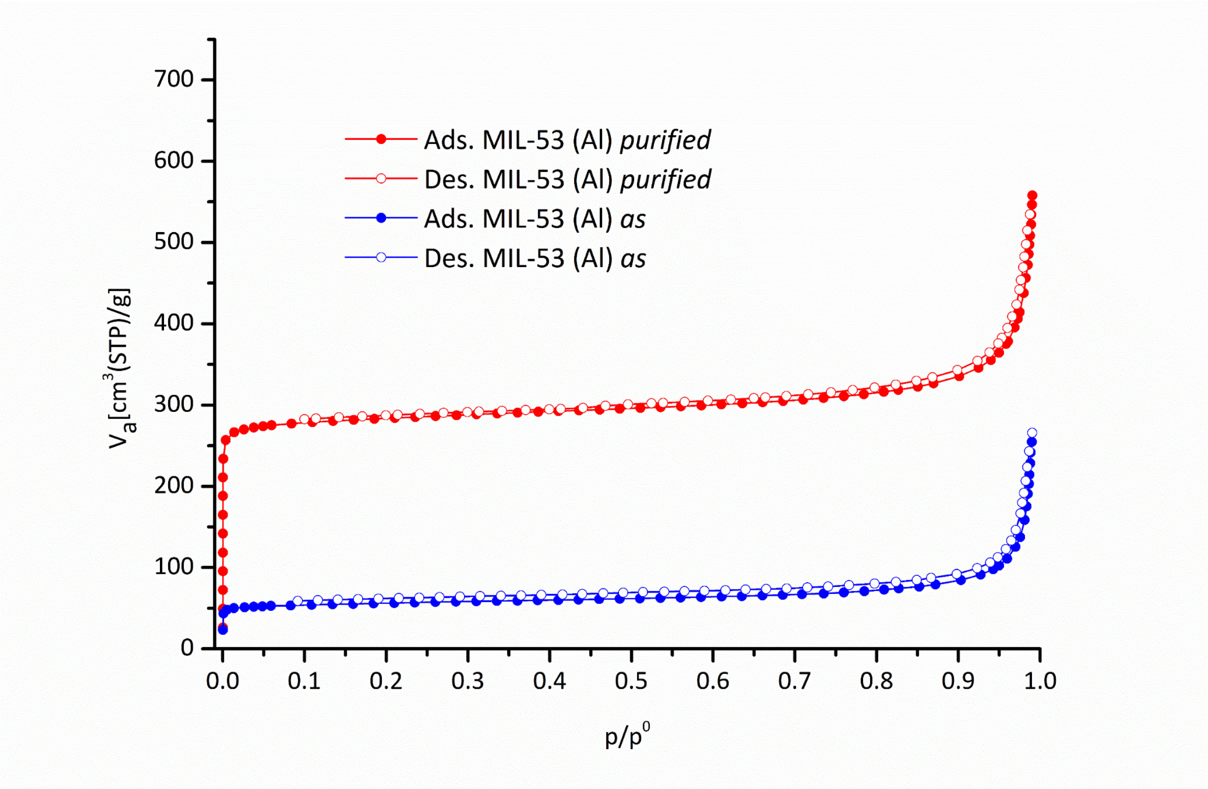

Figure S16. Nitrogen adsorption isotherm of Al-MIL-53 (activation: 220 °C under reduced pressure, 2 h) before (without preliminary ethanol treatment) and after purification. The as synthesized compound shows nearly no nitrogen uptake due to impurities like acetamide or residual metal species which are blocking the pores/decreasing the specific surface area.

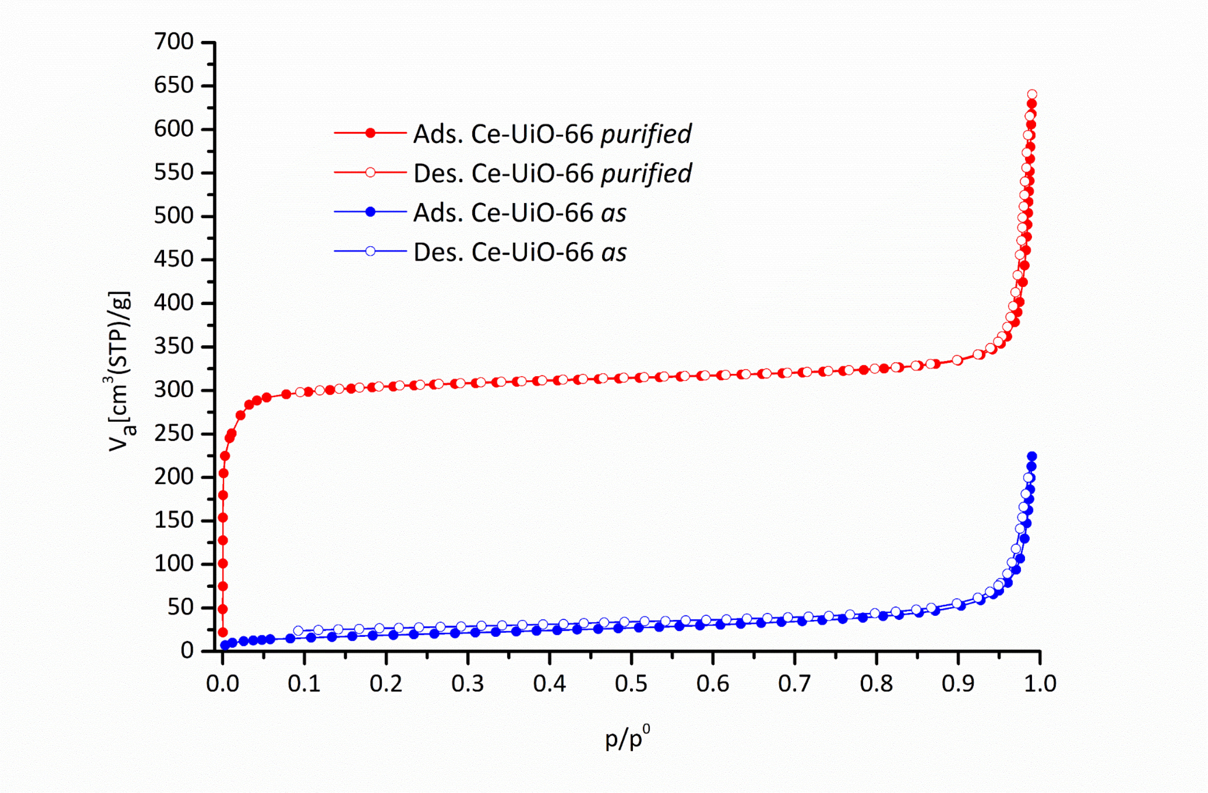

Figure S17. Nitrogen adsorption isotherm of Ce-UiO-66 (activation: 120 °C under reduced pressure, 16 h) before and after purification. The as synthesized compound shows nearly no nitrogen uptake due to impurities like acetamide or residual linker molecules which are blocking the pores/decreasing the specific surface area.

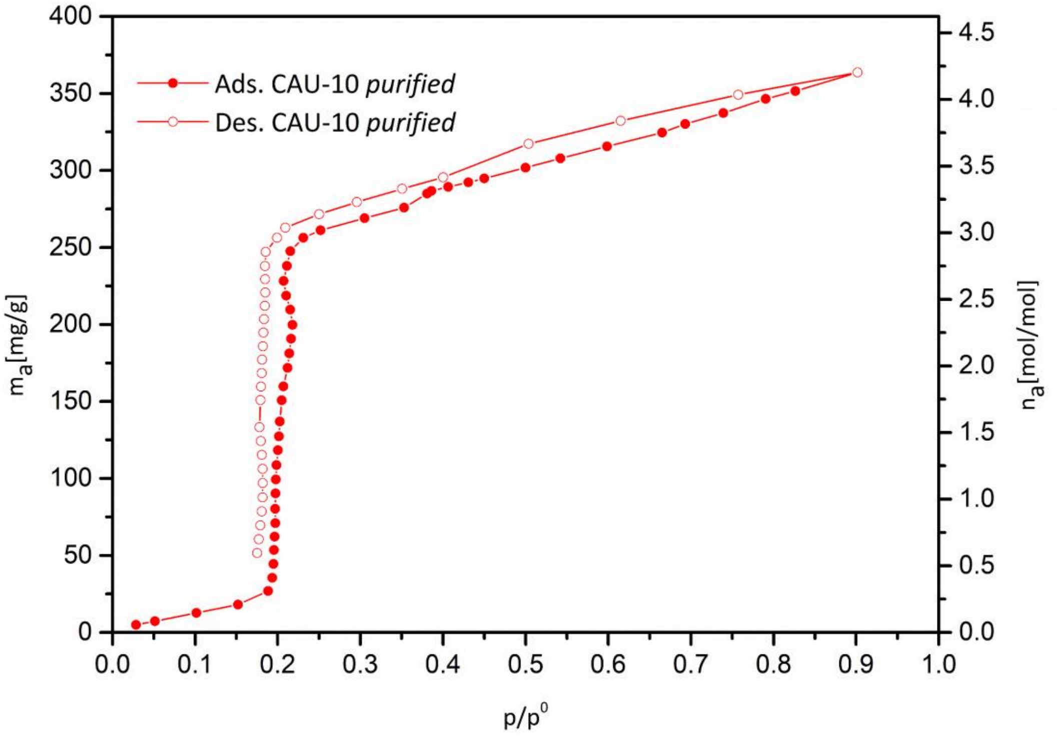

Figure S18. Water adsorption isotherm of CAU-10 (activation: 150 °C under reduced pressure, 2 h) after purification.

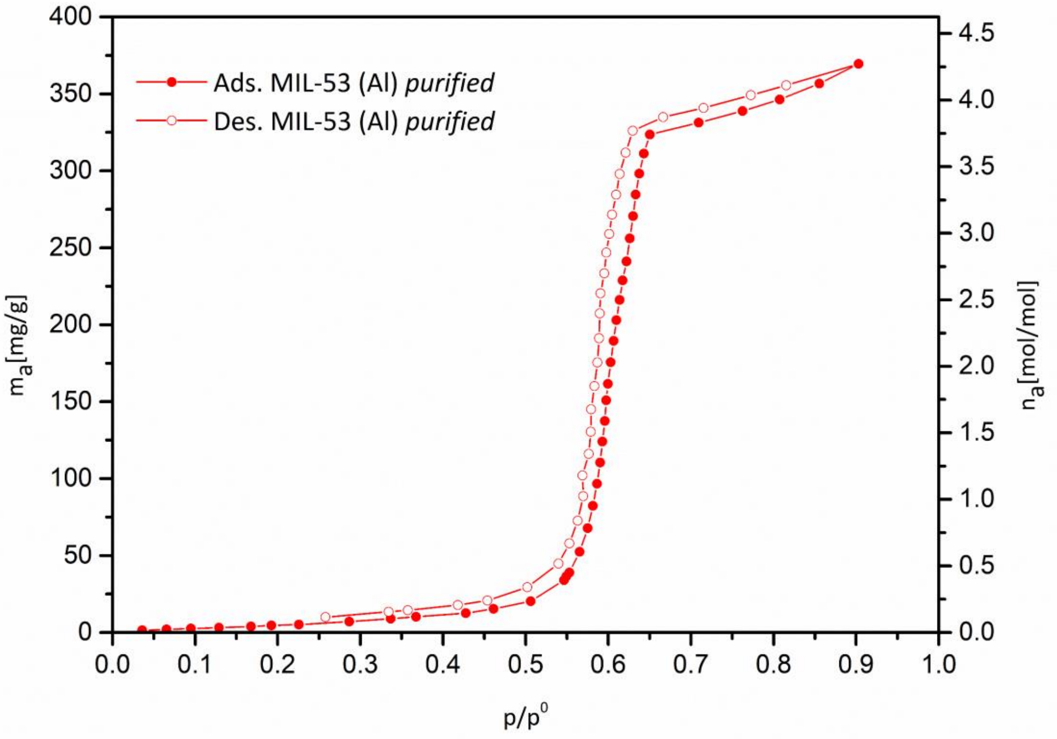

Figure S19. Water adsorption isotherm of Al-MIL-53 (activation: 220 °C under reduced pressure, 2 h) after purification.

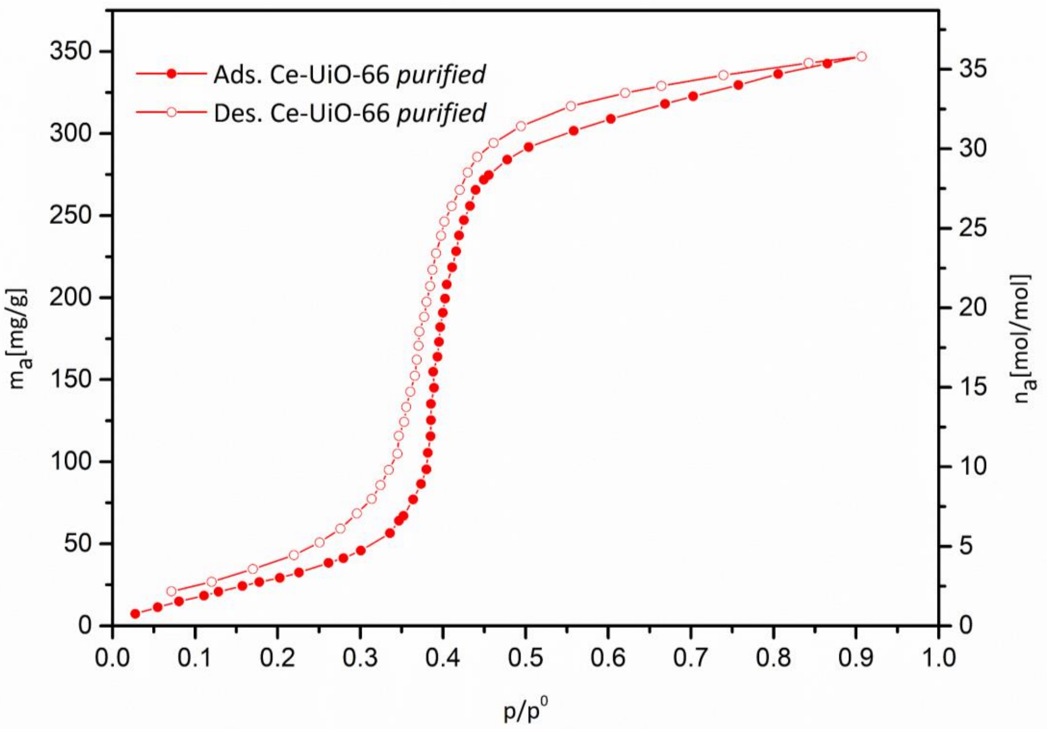

Figure S20. Water adsorption isotherm of Ce-UiO-66 (activation: 120 °C under reduced pressure, 16 h) after purification.

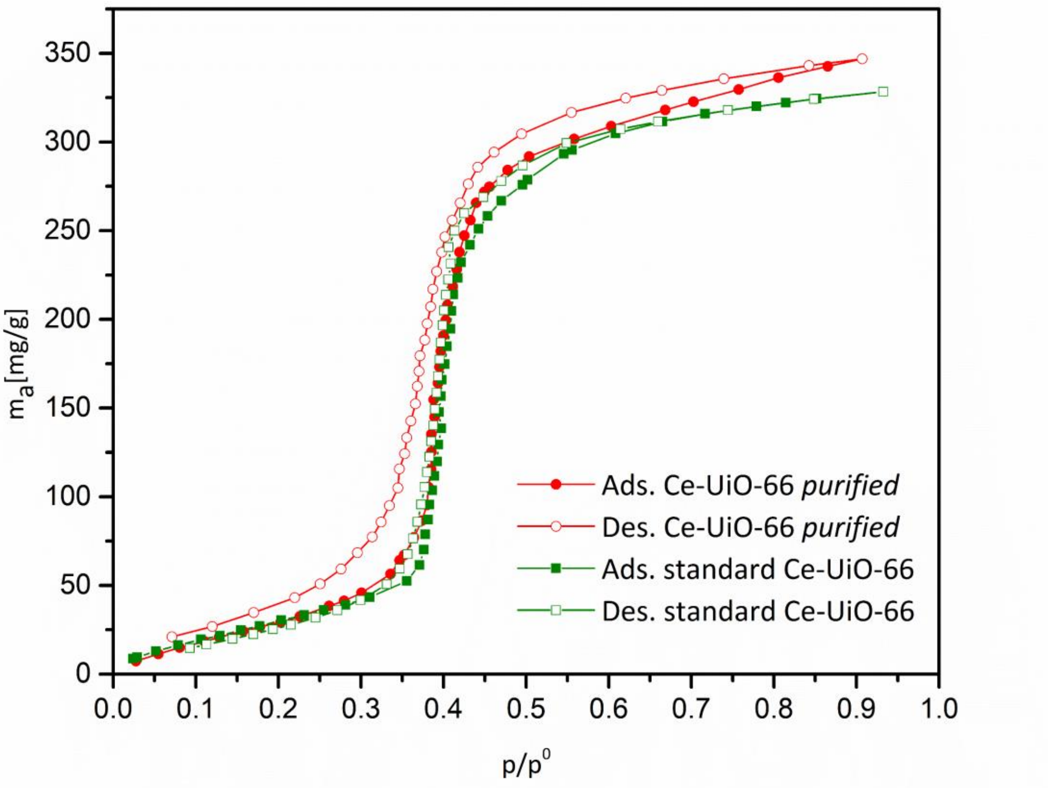

Figure S21. Water adsorption isotherms of Ce-UiO-66 after purification (this work) and standard Ce-UiO-66 in comparison (activation: 120 °C under reduced pressure, 16 h).

Infrared Spectroscopy

For characterization by infrared spectroscopy purified and as synthesized samples (see Synthetic Procedures) were used and compared. The comparison shows that purification is crucial for removing impurities like residual nitrate salts or acetamide, which is formed due to hydrolysis of the solvent (acetonitrile). After purification Al-MIL-53 still shows a weak IR band at 1702 cm^{-1} which can be attributed to protonated carboxylic acid groups. This is in line with a partially defective structure as observed via solid-state NMR spectroscopy. The intensity of the bands that can be assigned to water molecules and the terephthalate ions in Al-MIL-53 are also very small. Due to the low acetamide content (0.1 molecules per formula) in this sample, the corresponding vibrational bands for amides could not be clearly identified.

CAU-10

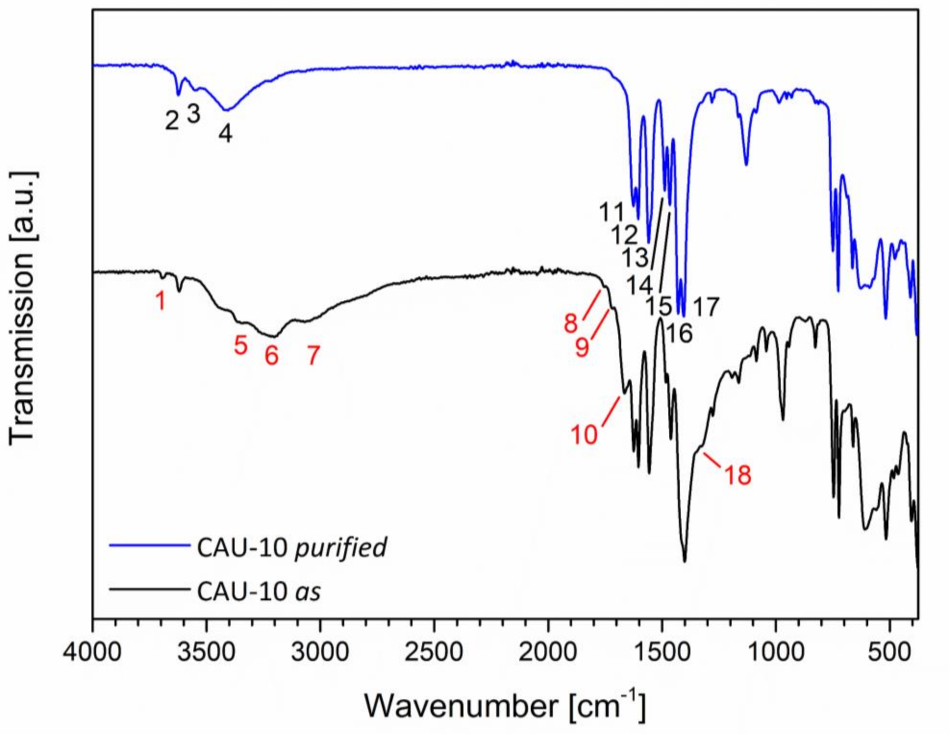

Figure S22. IR spectra of CAU-10 as synthesized and after purification.

Table S6. Important IR band assignments for CAU-10.

Label	Measured wavenumber [cm^{-1}]	Theoretical region [cm^{-1}]	Assignment
1	3693	3760-3000[24]	v(Al-O-H)
2	3625	3760-3000[24]	v(Al-O-H)
3	3551	3600-3200[24]	v(H-O-H) lattice water
4	3408	3550-3200[24]	v(H-O-H) hydrogen bonding, broad
5	3351	3375-3320[24]	v_{asym}(N-H) amides
6	3208	3205-3155[24]	v_{sym}(N-H) amides
7	3056	3300-3030[24]	v(N-H) ammonium chloride, broad
8	1751	1800-1740[24]	v(C=O) (free actic acid as monomers)
9	1714	1740-1700[24]	v(C=O) (free acetic acid, hydrogen bonded)
10	1667	1670-1650[24]	v(C=O) amide I band
11	1624	1695-1540[24,25]	v_{asym}(O-C-O) isophthalate ion
12	1603	1625-1590[24,25]	v(C=C) isophthalate ion
13	1556	1695-1540[24,25]	v_{asym}(O-C-O) isophthalate ion
14	1488	1590-1575[24,25]	v(C=C) isophthalate ion
15	1470	1525-1470[24,25]	v(C=C) isophthalate ion
16	1430	1470-1430[24,25]	v(C=C) isophthalate ion
17	1403	1440-1335[24,23] / 1430-1390[24]	v_{sym}(O-C-O) isophthalate ion / δ(N-H) ammonium chloride
18	1360	1410-1350[24]	$v_{asym}(NO_3)$, broad

Al-MIL-53

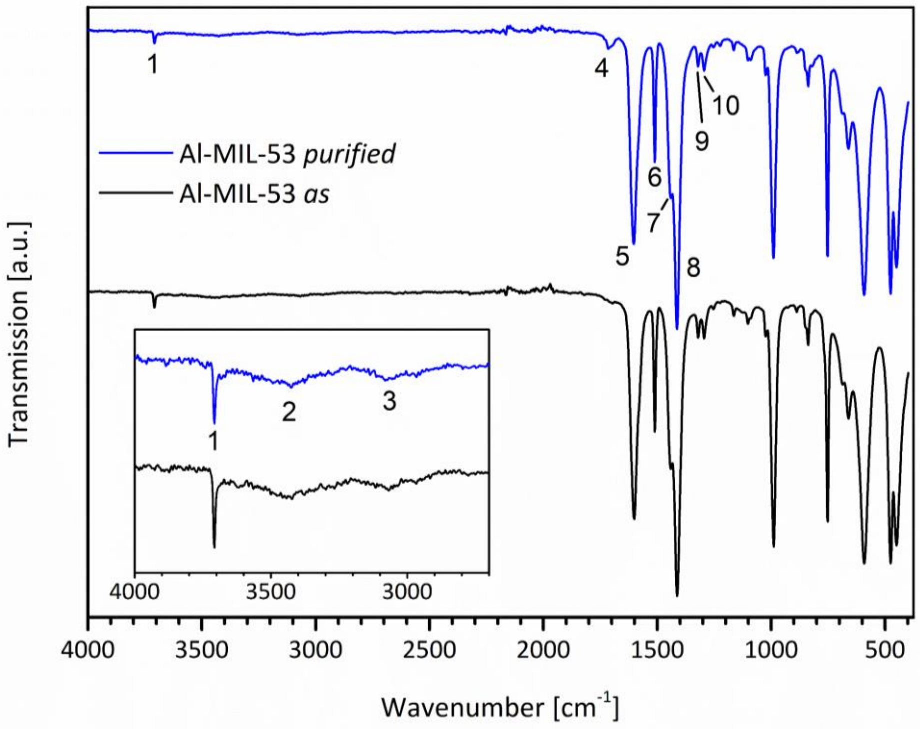

Figure S23. IR spectra of Al-MIL-53 as synthesized and after purification.

Table S7. Important IR band assignments for Al-MIL-53.

Label	Measured wavenumber [cm^{-1}]	Theoretical region [cm^{-1}]	Assignment
1	3708	3760-3000[24]	ν(Al-O-H)
2	3430	3550-3200[24]	ν(H-O-H), hydrogen bonding, broad
3	3066	3105-3000[24]	ν(C-H) aromatic protons, terephthalate ion
4	1702	1710-1680[24]	ν_{asym}(O-C-O) protonated terephthalic acid
5	1597	1695-1540[24,26]	ν_{asym}(O-C-O) terephthalate ion
6	1510	1525-1470[24,26]	ν(C=C) terephthalate ion
7	1442	1470-1430[24,26]	ν(C=C) terephthalate ion
8	1411	1440-1335[24,26]	ν_{sym}(O-C-O) terephthalate ion
9	1324	1330-1260[24]	δ(C=C) terephthalate ion
10	1293	1330-1260[24]	δ(C=C) terephthalate ion

Ce-UiO-66

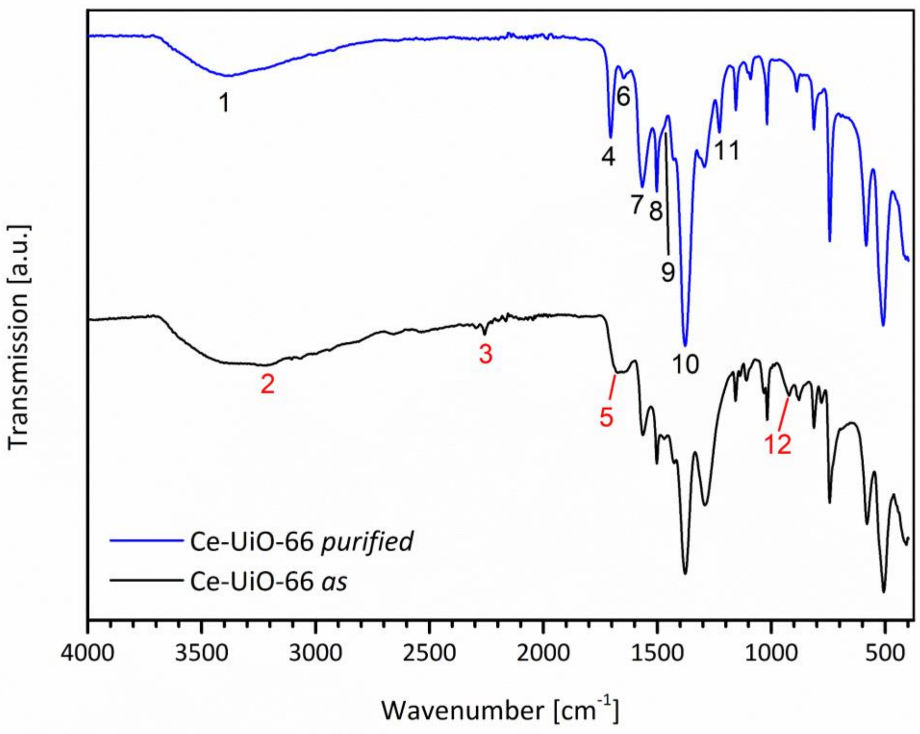

Figure S24. IR spectra of Ce-UiO-66 as synthesized and after purification.

Table S8. Important IR band assignments for Ce-UiO-66.

Label	Measured wavenumber [cm^{-1}]	Theoretical region [cm^{-1}]	Assignment
1	3400	3550-3200[24]	ν(H-O-H), hydrogen bonding, broad
2	3227	3375-3320[24]	$ν_{asym}$(N-H) amides
3	2258	2260-2230[24]	ν(C≡N) acetonitrile
4	1708	1730-1700[24]	ν(C=O) saturated methyl ketones, acetone
5	1682	1710-1660[24]	$ν_{sym}$(O-C-O) protonated terephthalic acid
6	1652	1670-1650[24]	ν(C=O) amide I band
7	1564	1695-1540[24,26]	$ν_{asym}$(O-C-O) terephthalate ion
8	1503	1525-1470[24,26]	ν(C=C) terephthalate ion
9	1472	1470-1430[24,26]	ν(C=C) terephthalate ion
10	1376	1440-1335[24,26]	$ν_{sym}$(O-C-O) terephthalate ion
11	1228	1325-1215[24]	ν(C-C) aliphatic ketones, acetone
12	922	960-875[24]	δ(O-H) out-of-plane, broad, free terephthalic acid

Scanning (SEM) & Transmission Electron Microscopy (TEM)

In order to examine the morphology and to estimate the particle size magnitude of Al-MIL-53, scanning and transmission electron microscopy micrographs were collected. A purified sample was used (see Synthetic Procedures). The particles are very small and strongly intergrown. The observed particle size magnitude is in line with the average particle size of 140 to 270 nm as determined via mercury intrusion (Mayer-Stowe method).

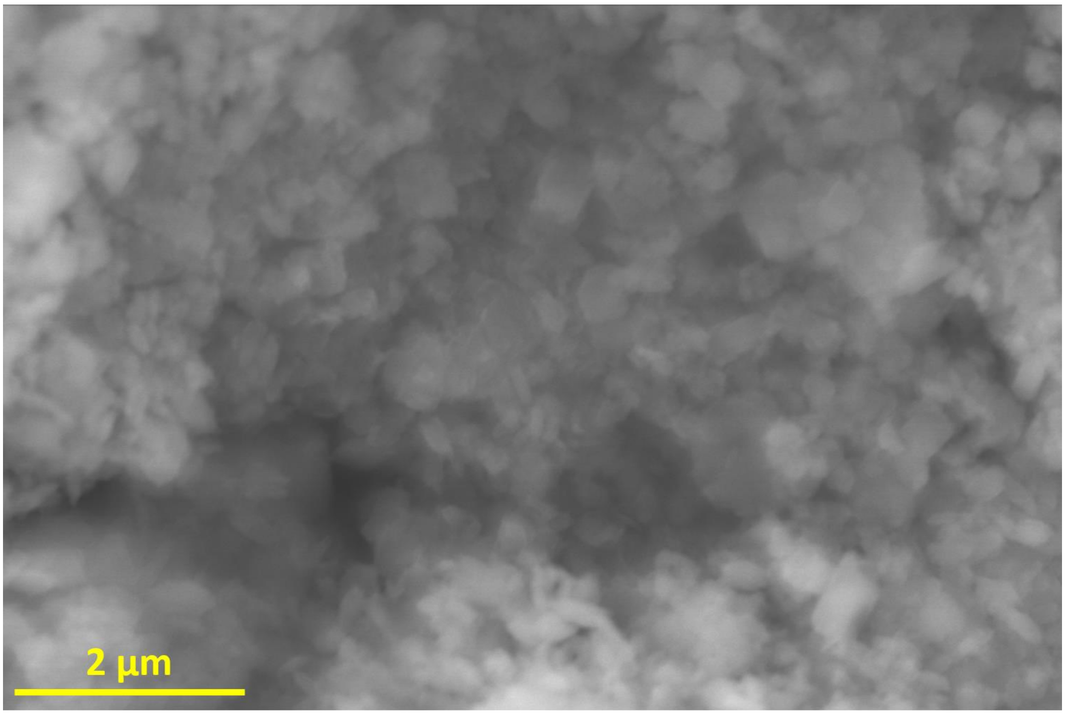

Figure S25. SEM micrograph of Al-MIL-53 particles collected at 20 kV.

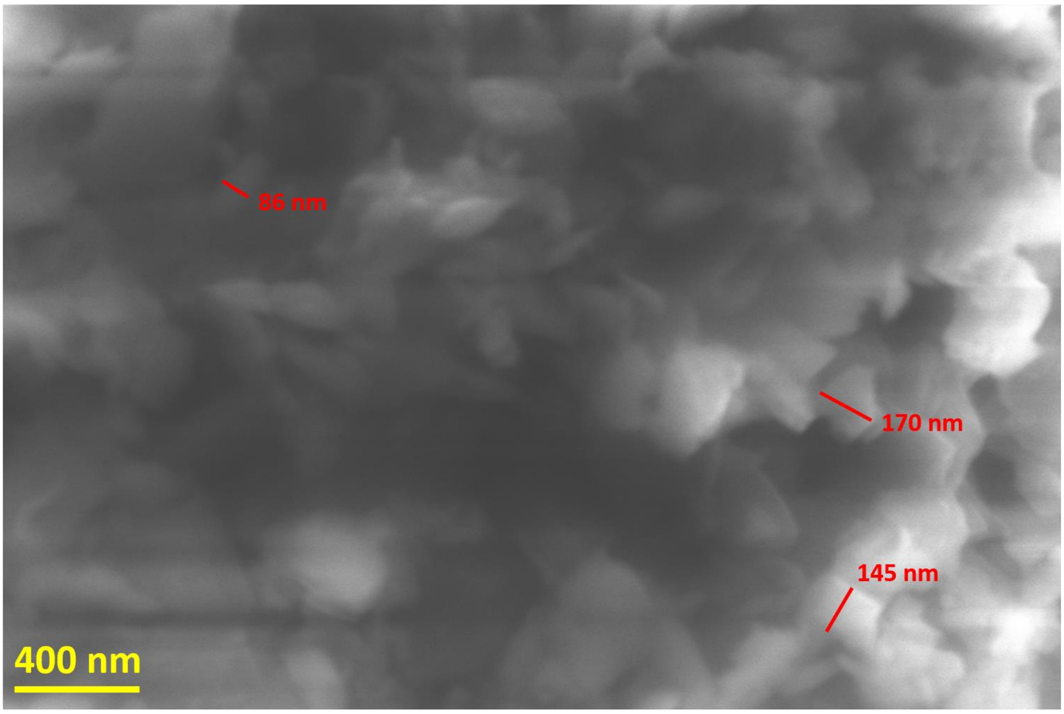

Figure S26. SEM micrograph of Al-MIL-53 particles collected at 5 kV including diameter measurements for some particles (red slices and corresponding values).

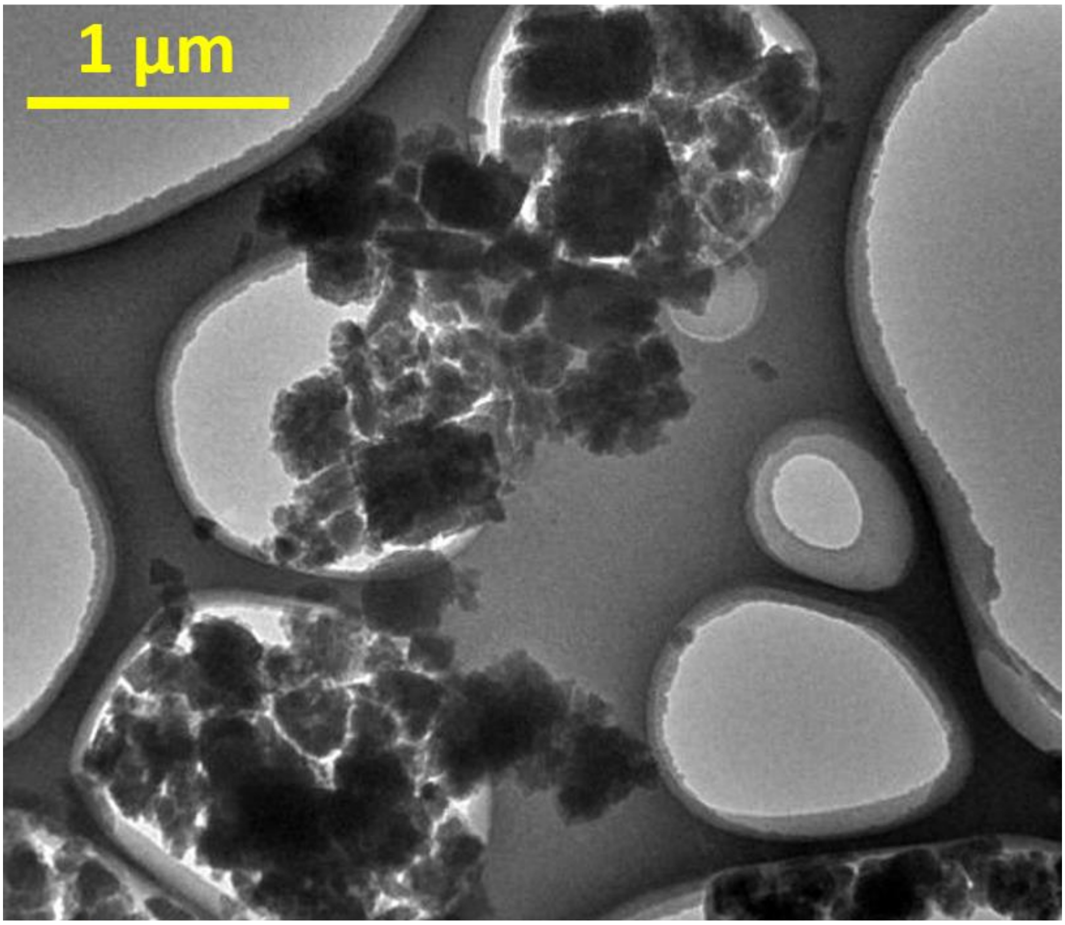

Figure S27. Bright-field TEM micrograph of Al-MIL-53 particles on a holey carbon grid.

Solid-State NMR Spectroscopy

Supporting tables and figures for the solid-state NMR studies are given in the following.

Table S9. Refinement parameters of the deconvolution of ^{1}H spectrum. G/L describes the ratio between Gaussian and Lorentzian profile. Completely Gaussian profile with G/L = 1, completely Lorentzian profile at G/L = 0.

δ_{iso} [ppm]	G/L	FWHM / ppm	Integral
1.55	1	1.51	0.27
2.65	0.55	0.56	1
3.95	1	1.34	0.33
6.51	1	2.3	0.48
8.03	0.43	0.62	3.98
12.11	1	1.49	0.13

Table S10. Refinement parameters of the deconvolution of the ^{27}Al MAS NMR spectrum. Typical lineshapes for second order quadrupolar coupling interaction were used, including gaussian distribution of the quadrupolar coupling constant C_q. $\sigma(C_q)$ represents the variance of the gaussian distribution.

Parameter	Defect-free Al-MIL-53	Defective Al-MIL-53
δ_{iso} [ppm]	2.0	9.6
C_q [MHz]	8.8	6.0
$\sigma(C_q)$ [MHz]	1.2	1.0
η	0	0
Guassian Broadening [Hz]	800	1200
Relative Intensity [%]	84.0	16.0

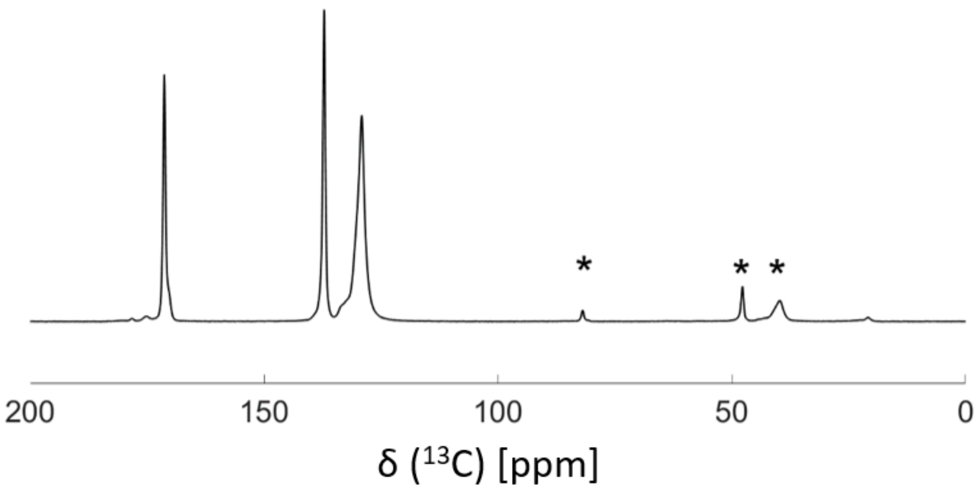

Figure S28. ^{13}C CPMAS spectrum at spinning speed of 9 kHz. Rotational sidebands are denoted by an asterisk.

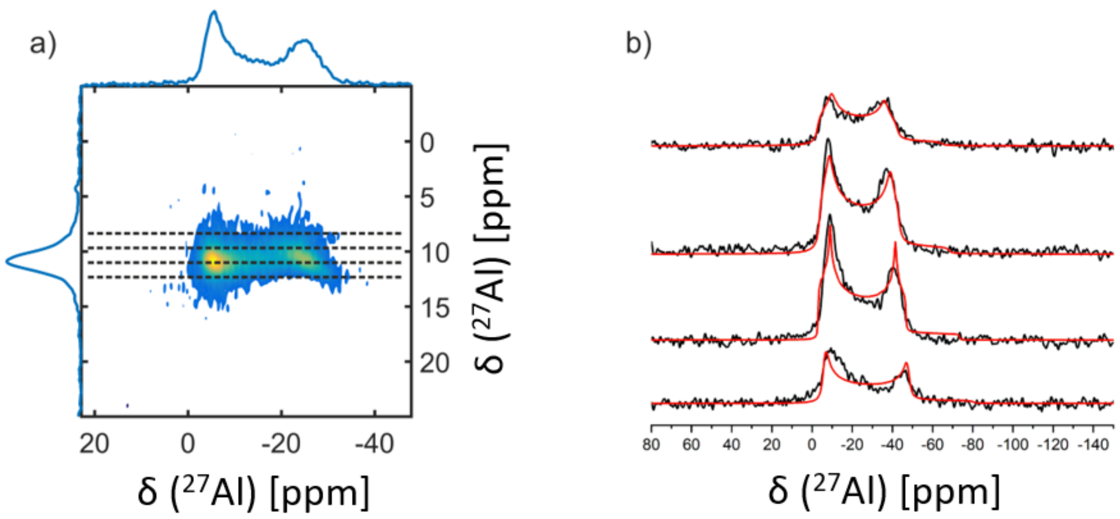

Figure S29. a) ^{27}Al STMAS spectrum, revealing a distribution of quadrupolar coupling constants. b) profile fits of the 1-D rows.

References

[1] I. Beurroies, M. Boulhout, P. L. Llewellyn, B. Kuchta, G. Ferey, C. Serre, R. Denoyel, *Angew. Chem. Int. Ed.* **2010**, *49*, 7526-7529.

[2] S. Henke, M. T. Warmby, G. Kieslich, I. Hante, H. Schneemann, Y. Wu, D. Daisenberg, A. K. Cheetham, *Chem. Sci.* **2018**, *9*, 1654-1660.

[3] P. Ramaswamy, J. Wieme, E. Alvarez, L. Vanduyfhuys, J.-P. Itié, P. Fabry, V. Van Speybroeck, C. Serre, P. G. Yot, G. Maurin, *J. Mater. Chem. A* **2017**, *5*, 1104711054.

[4] P. G. Yot, Q. Ma, J. Haines, Q. Yang, A. Ghoufi, T. Devic, C. Serre, V. Dmitriev, G. Férey, C. Zhong, G. Maurin, *Chem. Sci.* **2012**, *3*, 1100-1104.

[5] P. G. Yot, Z. Boudene, J. Macia, D. Granier, L. Vanduyfhuys, T. Verstraelen, V. Van Speybroeck, T. Devic, C. Serre, G. Férey, N. Stock, G. Maurin, *Chem. Commun.* **2014**, *50*, 9462-9464.

[6] P. G. Yot, L. Vanduyfhuys, J. Rodriguez, Elsa Alvarez, J.-P. Itiè, P. Fabry, N. Guillou, T. Devic, I Beurroies, P. L. Llewellyn, V. Van Speybroeck, C. Serre, G. Maurin, *Chem. Sci.* **2016**, *7*, 446-450.

[7] M. Wahiduzzaman, J.-P. Itié, N. Stock, G. Maurin, P. G. Yot, *Polyhedron* **2018**, *155*, 144.

[8] P. G. Yot, M. Wahiduzzaman, E. Elkaim, P. Fertey, P. Fabry, C. Serre, G. Maurin, *Dalton Trans.* **2019**, *48*, 1656.

[9] T. F. Kemp, M. E. Smith, *Solid State Nucl. Magn. Reson.* **2009**, *35*, 243-252.

[10] A. Pines, M. G. Gibby, J. S. Waugh, *J. Chem. Phys.* **1972**, *56*, 1776-1777.

[11] B. M. Fung, A. K. Khitrin, K. Ermolaev, *J. Magn. Reson.* **2000**, *142*, 97-101.

[12] H. T. Kwak, Z. Gan, *J. Magn. Reason.* **2003**, *164*, 369-372.

[13] R. Siegel, J. Rocha, L. Mafra, *Chem. Phys. Lett.* **2009**, *470*, 337-341.

[14] M. Lammert, M. T. Wharmby, S. Smolders, B. Bueken, A. Lieb, K. A. Lomachenko, D. De Vos, N. Stock, *Chem. Commun.* **2015**, *51*, 12578.

[15] T. Loiseau, C. Serre, C. Huguenard, G. Fink, F. Taulelle, M. Henry, T. Bataille, G. Férey, *Chem. Eur. J.* **2004**, *10*, 1373-1382.

[16] K. Barthelet, J. Marrot, D. Riou, G. Férey, *Angew. Chem. Int. Ed.* **2002**, *41*, 281-284.

[17] D. Fröhlich, E. Pantatosaki, P. D. Kolokathis, K. Markey, H. Reinsch, M. Baumgartner, M. A. van der Veen, D. E. De Vos, N. Stock, G. K. Papadopoulos, S. K. Henninger, C. Janiak, *J. Mater. Chem. A* **2016**, *4*, 11859.

[18] J. H. Cavka, S. Jakobsen, U. Olsbye, N. Guillou, C. Lamberti, S. Bordiga, K. P. Lillerud, *J. Am. Chem. Soc.* **2008**, *130*, 13850-13851.

[19] V. Guillerm, F. Ragon, M. Dan-Hardi, T. Devic, M. Vishnuvarthan, B. Campo, A. Vimont, G. Clet, Q. Yang, G. Maurin, G. Férey, A. Vittadini, S. Gross, C. Serre*, Angew. Chem.* **2012**, *124*, 9401-9405.

[20] H. Furukawa, F. Gándara, Y.-B. Zhang, J. Jiang, W. L. Queen, M. R. Hudson, O. M. Yaghi, *J. Am. Chem. Soc.* **2014**, *136*, 4369-4381.

[21] M. J. Cliffe, W. Wan, X. Zou, P. A. Chater, A. K. Kleppe, M. G. Tucker, H. Wilhelm, N. P. Funnell, F.-X. Coudert, A. L. Goodwin, *Nat. Commun.* **2014**, *5*, 4176.

[22] S. S.-Y. Chui, S. M.-F. Lo, J. P. H. Charmant, A. G. Orpen, I. D. Williams, *Science* **1999**, *283*, 1148-1150.

[23] S. R. Miller, P. A. Wright, C. Serre, T. Loiseau, J. Marrot, G. Férey, Chem. Commun. 2005, 3850-3852.

[24] G. Socrates, *Infrared and Raman Characteristic Group Frequencies: Tables and Charts, Vol. 3*, Wiley & Sons Ltd., Chichester, **2001**.

[25] F. Bardak, C. Karaca, S. Bilgili, A. Atac, T. Mavis, A. M. Asiri, M. Karabacak, E. Kose, *Spectrochim. Acta A* **2016**, *165*, 33-46.

[26] J. M. Salazar, G. Weber, J. M. Simon, I. Bezverkhyy, J. P. Bellat, *J. Chem. Phys.* **2015**, *142*, 124702.
